# Age trends of internalizing and externalizing problems in children with neurofibromatosis type 1: a multicenter study

**DOI:** 10.1007/s00787-026-03039-x

**Published:** 2026-04-29

**Authors:** Dan Liu, Xiaoli Zong, Jonathan M. Payne, Peter L. Stavinoha, Pamela L. Wolters, Karin S. Walsh, Jennifer Janusz, Bonita P. Klein-Tasman, Stephanie M. Morris, Carrie E. Bearden, Natalie Pride, Yang Hou

**Affiliations:** 1 Florida State University, Tallahassee, FL USA; 2 Army Medical University, Chongqing, China; 3 Murdoch Children’s Research Institute, Melbourne, Australia; 4 Department of Paediatrics, University of Melbourne, Melbourne, Australia; 5 University of Texas MD Anderson Cancer Center, Houston, TX USA; 6 Pediatric Oncology Branch, Center for Cancer Research, National Cancer Institute, National Institutes of Health, Bethesda, MD USA; 7 Children’s National Hospital, Washington, DC USA; 8 The George Washington University School of Medicine, Washington, DC USA; 9 Children’s Hospital Colorado, Aurora, CO USA; 10 University of Wisconsin-Milwaukee, Milwaukee, WI USA; 11 Kennedy Krieger Institute, Baltimore, MD USA; 12 University of California, Los Angeles, CA USA; 13 Children’s Hospital at Westmead, Sydney, Australia; 14 Department of Behavioral Sciences and Social Medicine, College of Medicine, Florida State University, 1115 West Call Street, Tallahassee, FL 32306-4300 USA

**Keywords:** Age trends, Internalizing, Externalizing, Neurofibromatosis type 1, Pediatric rare disease, ADHD

## Abstract

**Purpose:**

Children with neurofibromatosis type 1 (NF1) frequently experience internalizing and externalizing problems, yet their developmental patterns remain poorly understood. This study examined age-related trends and subgroup differences in these symptoms.

**Methods:**

Integrative data analysis combined 1,841 observations from 1,194 children with NF1 (ages 3–18) across nine institutions. Internalizing/externalizing problems were assessed using parent-rated Child Behavior Checklist (CBCL sample; *n* = 1,087 observations) or Behavior Assessment System for Children (BASC sample; *n* = 754 observations). The two samples were analyzed separately. Time-varying effect modeling characterized age-related symptom patterns overall and across subgroups defined by sex, NF1 inheritance mode, the presence of plexiform neurofibromas, and attention-deficit/hyperactivity disorder (ADHD) diagnosis.

**Results:**

Across both assessments, children with NF1 exhibited greater internalizing and externalizing problems relative to normative means, although mean scores generally remained below clinical thresholds. Deviations were greater in older than younger children for internalizing problems, whereas deviations in externalizing symptoms remained relatively similar across age. Age patterns for both problems were largely consistent across subgroups except that children with ADHD showed higher symptom levels and distinct age patterns.

**Conclusions:**

Findings highlight age-related differences in behavioral symptoms in NF1 relative to population norms, with greater internalizing deviations and relatively consistent externalizing deviations across age, as well as more pronounced deviations among children with co-occurring ADHD. Although interpretation is limited by reliance on parent reports, cross-sectional data, and normative rather than control comparisons, findings underscore the importance of ongoing monitoring and future longitudinal, multi-informant research to clarify developmental trajectories and clinical needs in NF1.

**Supplementary Information:**

The online version contains supplementary material available at https://doi.org/10.1007/s00787-026-03039-x.

## Introduction

Mental health concerns are often overlooked in children with rare diseases, as clinical attention focuses primarily on physical health [1]. Consequently, these children may not receive adequate mental health support, despite evidence linking rare diseases to elevated emotional and behavioral challenges [2]. One such condition is neurofibromatosis type 1 (NF1), an autosomal dominant genetic disorder affecting about 1 in 3,000 individuals [3]. NF1 is characterized by tumors (e.g., plexiform neurofibromas [PNs]), skin pigmentation changes, bone defects, and heightened risk for cognitive and neurodevelopmental challenges [3–7]. Psychosocial difficulties are common in children with NF1, impacting quality of life and increasing long-term psychiatric risk [8, 9], making early identification and intervention critical.

Most NF1 research focuses on neurodevelopmental conditions like attention-deficit/hyperactivity disorder (ADHD) [10] and autism spectrum disorder (ASD) [11], with less attention to other psychiatric difficulties, including internalizing (e.g., anxiety, depression, somatic complaints) and externalizing problems (e.g., aggression, irritability, delinquency) [8, 12, 13], two core domains of mental health. Evidence from meta-analyses and diagnostic studies shows elevated internalizing and externalizing symptoms in youth with NF1 relative to typically developing peers [14, 15], but developmental trajectories remain poorly characterized.

In the general population, internalizing problems typically increase from childhood to adolescence [16], while externalizing problems rise through middle childhood and peak in mid-adolescence before declining [17]. Whether similar developmental patterns occur among children with NF1 remains unclear. Prior studies have reported little evidence of age-related change, although these findings were likely constrained by small sample sizes and limited developmental coverage [18]. NF1-specific factors—including progressive physical symptoms [3], learning difficulties [19], and comorbidities—may alter trajectories, highlighting the need for developmental investigation.

Another underexplored area is how specific factors shape the developmental patterns of internalizing and externalizing problems in children with NF1. Variation in symptom trajectories may reflect effects of sex, with preliminary evidence suggesting females with NF1 face elevated psychiatric risk [9, 20]; inheritance mode (familial vs. sporadic NF1), for which findings remain mixed [21, 22]; presence and progression of plexiform neurofibromas (PNs), which may increase disease burden and psychosocial stress [23]; and comorbid ADHD, which can exacerbate internalizing and externalizing symptoms [24–26].

A major limitation of prior NF1 studies is the reliance on small, single-site samples, with over 90% including fewer than 100 participants, and only 5% using longitudinal designs [14]. Such constraints reduce statistical power, limit generalizability, and hinder examination of complex developmental patterns. To address these gaps and strengthen the robustness and generalizability of our findings, the current study used two large, harmonized samples from nine sites and two standardized tools—one sample assessed with the Child Behavior Checklist (CBCL) [27] and the other with the Behavior Assessment System for Children (BASC) [28, 29]. While both instruments provide valid assessments of internalizing and externalizing problems, they differ in subscale composition and item content, and thus separate analyses were conducted for each instrument to avoid imposing assumptions of measurement equivalence across samples and to generate measure-specific estimates. Using time-varying effect modeling (TVEM) [30], we explored two research objectives: (1) age-related variation in internalizing and externalizing problems in children with NF1, and (2) whether the age patterns differ by sex, NF1 inheritance mode, the presence of PNs, and ADHD diagnosis.

## Methods

### Participants and procedure

This study included primarily cross-sectional data from 1,841 observations from 1,194 children with NF1, drawn from two independent samples across nine institutions in the United States and Australia. Sample 1 (*n* = 1,087 observations from 693 children with NF1), assessed with CBCL, was used for initial analysis, while Sample 2 (*n* = 754 observations from 501 children with NF1), assessed with BASC, served as a replication sample. De-identified individual-level data were harmonized following integrative data analysis (IDA) guidelines [31, 32]. IDA can enhance statistical power, sample heterogeneity, representativeness, and age range [33]. Analyses were conducted at the observation level; reported sample sizes (*n*) reflect the number of observations unless noted otherwise. Institutional review boards (IRB) approved data collection at each site. About half the sites obtained written consent from participants or their guardians; others utilized retrospectively collected clinical data. The IRBs of University of Kentucky (ID 67554) and Florida State University (ID STUDY00003513) approved the IDA.

### Measures

#### Outcomes

Internalizing and externalizing problems were assessed with parent-rated CBCL [27] in Sample 1 (the CBCL Sample) and BASC [28, 29] in Sample 2 (the BASC Sample). Total internalizing and externalizing scores were the focal outcomes. Subdomains (Tables 1 and 2) were included in supplementary analyses. For each participant, the standardized T scores (*M*_norm_ = 50, *SD*_norm_ = 10) provided by the respective instrument at each site were used directly in analyses without additional aggregation or transformation; higher scores indicate greater symptom severity. Both CBCL and BASC demonstrate good reliability and validity with high converging validity between the two measures [34, 35].

**Table 1:** Characteristics of each site and the combined sample that provided CBCL data (Sample 1)

Characteristic	University of Texas, MD Anderson Cancer Center, USA	Children’s National Hospital, USA	University of California, Los Angeles, USA	Murdoch Children’s Research Institute, Australia	Children’s Hospital at Westmead, Australia	Children’s Hospital Colorado, USA	Combined
Sample	Clinical referrals through the NF1 clinic	Convenience sample recruited through patients seen for clinical evaluation in a pre-clinical appointment survey from a pediatric neuropsychology clinic	Referrals from NF1 clinics across Southern California and lab website	Convenience sample recruited on a sequential basis from a multidisciplinary NF1 Clinic	Convenience sample recruited on a sequential basis from a neurogenetics clinic	Convenience sample recruited through patients seen for outpatient neuropsychological evaluation	-
Measure	CBCL/4–18, 1.5-5, 6–18	CBCL/1.5-5, 6–18	CBCL/6–18	CBCL/6–18	CBCL/1.5-5, 6–18	CBCL/1.5-5, 6–18	-
Total *n*	637	214	83	72	49	32	1087
Internalizing problems *n*	632	187	83	72	48	32	1054
Anxious/depressed *n*	635	214	82	72	49	32	1084
Withdrawn/depressed *n*	611	189	83	72	49	25	1029
Somatic complaints *n*	635	213	83	71	49	32	1083
Externalizing problems *n*	635	187	83	72	48	32	1057
Rulebreaking behaviors n	610	189	83	72	48	25	1027
Aggressive behaviors *n*	635	213	83	72	49	25	1077
Age, median (range), yrs	11.10 (3–18)	9.58 (3–18)	12.92 (7–18)	10.13 (6–15)	9.92 (3–16)	10.56 (3–18)	10.83 (3–18)
Sex *n*	637	210	83	71	49	32	1082
Male *n* (%)	352 (55.3)	114 (54.3)	34 (41.0)	41 (57.7)	23 (46.9)	16 (50.0)	580 (53.6)
Female *n* (%)	285 (44.7)	96 (45.7)	49 (59.0)	30 (42.3)	26 (53.1)	16 (50.0)	502 (46.4)
NF1 inheritance mode *n*	610	0	0	72	46	30	758
Sporadic *n* (%)	367 (60.2)	0	0	48 (66.7)	27 (58.7)	14 (46.7)	456 (60.2)
Familial *n* (%)	243 (39.8)	0	0	24 (33.3)	19 (41.3)	16 (53.3)	302 (39.8)
Plexiform neurofibromas n	637	0	0	51	12	31	731
No *n* (%)	430 (67.5)	0	0	39 (76.5)	9 (75.0)	29 (93.5)	507 (69.4)
Yes *n* (%)	207 (32.5)	0	0	12 (23.5)	3 (25.0)	2 (6.5)	224 (30.6)
ADHD diagnosis *n*	0	213	83	65	34	32	427
No *n* (%)	0	89 (41.8)	43 (51.8)	57 (87.7)	25 (73.5)	20 (62.5)	234 (54.8)
Yes *n* (%)	0	124 (58.2)	40 (48.2)	8 (12.3)	9 (26.5)	12 (37.5)	193 (45.2)

*n* valid number of observations. *NF1* neurofibromatosis type 1. *CBCL* Child Behavior Checklist. *ADHD* attention-deficit/hyperactivity disorder

**Table 2:** Characteristics of each site and the combined sample that provided BASC data (Sample 2)

Characteristic	National Cancer Institute, USA	Children’s Hospital Colorado, USA	University of Wisconsin–Milwaukee, USA	Washington University in St. Louis, USA	Children’s Hospital at Westmead, Australia	University of Texas, MD Anderson Cancer Center, USA	Combined
Sample	Participants in an NF1 natural history study recruited nationally (clinicaltrial.gov number: NCT00924196)	Convenience sample recruited through patients seen for outpatient neuropsychological evaluation	Convenience sample from NF1 clinic	Convenience sample from NF1 outpatient clinic	Convenience sample recruited on a sequential basis from a neurogenetics clinic	Clinical referrals through the NF1 clinic	-
Measure	BASC 2	BASC 2, BASC 3	BASC 2	BASC 2	BASC 3	BASC 3	-
Total *n*	285	179	172	99	12	7	754
Internalizing problems *n*	285	179	172	99	12	7	754
Anxiety *n*	285	176	172	99	12	7	754
Depression *n*	285	176	172	99	12	7	754
Somatization *n*	285	176	172	98	12	7	754
Externalizing problems *n*	285	178	172	99	12	7	753
Conduct problems *n*	232	150	97	84	12	7	582
Aggression *n*	285	175	172	99	12	7	750
Age, median (range), yrs	11.82 (3–18)	10.59 (3–18)	6.33 (3–13)	9 (3–17)	11.63 (6–13)	13.88 (13–16)	9.59 (3–18)
Sex *n*	285	179	172	99	12	7	754
Male *n* (%)	165 (57.9)	84 (46.9)	105 (61.0)	52 (52.5)	7 (58.3)	2 (28.6)	415 (55.0)
Female *n* (%)	120 (42.1)	95 (53.1)	67 (39.0)	47 (47.5)	5 (41.7)	5 (71.4)	339 (45.0)
NF1 inheritance mode *n*	264	175	169	98	6	6	718
Sporadic *n* (%)	159 (60.2)	122 (69.7)	112 (66.3)	51 (52.0)	4 (66.7)	3 (50.0)	451 (62.8)
Familial *n* (%)	105 (39.8)	53 (30.3)	57 (33.7)	47 (48.0)	2 (33.3)	3 (50.0)	267 (37.2)
Plexiform neurofibromas *n*	235	179	38	0	6	7	465
No *n* (%)	25 (10.6)	147 (82.1)	26 (68.4)	0	5 (83.3)	5 (71.4)	208 (44.7)
Yes *n* (%)	210 (89.4)	32 (17.9)	12 (31.6)	0	1 (16.7)	2 (28.6)	257 (55.3)
ADHD diagnosis	284	179	0	0	12	0	475
No *n* (%)	197 (69.4)	131 (73.2)	0	0	8 (66.7)	0	295 (62.1)
Yes *n* (%)	87 (30.6)	48 (26.8)	0	0	4 (33.3)	0	180 (37.9)

*n* valid number of observations. *NF1* neurofibromatosis type 1. *BASC* Behavior Assessment System for Children. *ADHD* attention-deficit/hyperactivity disorder

#### Covariates

Covariates were reported by parents/primary caregivers and/or retrieved from medical records and harmonized across sites through recoding procedures (Online Resource 1: eTables 1–2). Demographic covariates included age and biological sex (male/female). NF1-specific covariates included inheritance mode (sporadic vs. familial) and the presence of PNs (no/yes). ADHD diagnosis (no/yes) was included as a clinical covariate. Because standardized diagnostic interviews were not uniformly administered across sites, ADHD status reflects clinically recognized or parent-reported ADHD rather than a gold-standard diagnostic assessment.

### Statistical analysis

TVEM was used to characterize age-related patterns of internalizing and externalizing problems in the total samples and by subgroups based on demographic, NF1 disease-related, and clinical factors. TVEM is a non-parametric technique suitable for modeling smooth developmental trends over time or across ages without assuming predefined functional forms (e.g., linear or quadratic) [30, 36]. Though developed for intensive longitudinal data, it is adaptable to cross-sectional age-based designs [19, 37]. In this study, TVEM was applied to estimate coefficients flexibly, allowing for the construction of smooth age-related trajectories. A participant-level random intercept was included to account for within-person correlation due to repeated observations.

**Fig. 1:**
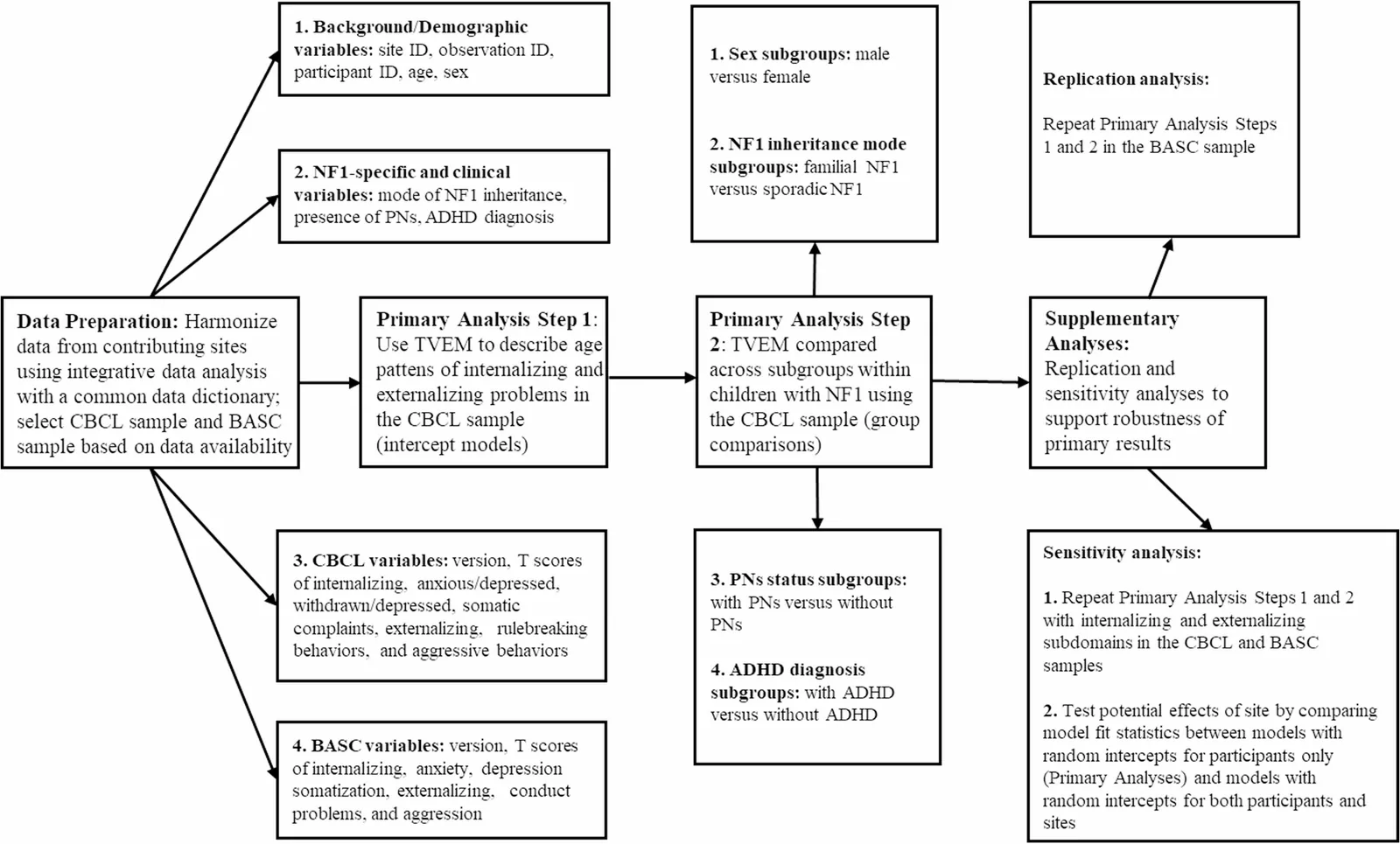
Analytic pipeline overview. Note. NF1 = neurofibromatosis type 1; PNs = plexiform neurofibromas; ADHD = attention-deficit/hyperactivity disorder; CBCL = Child Behavior Checklist; BASC = Behavior Assessment System for Children; TVEM = time varying effect modeling

Main analyses proceeded in two steps (Fig. 1): (1) intercept-only models examined general age patterns; (2) stratified models assessed subgroup differences. TVEM estimates the intercepts with 95% confidence intervals (CIs) for each age. Deviations from normative means (T = 50) were significant if 95% CIs did not include 50 [19, 37]. Significant age or subgroup differences were indicated by non-overlapping 95% CIs between specific age points or groups. In this study, TVEM coefficients were estimated using the B-spline approach [36], with a SAS macro [38], which excludes cases with missing data per model. Analyses were conducted with the CBCL Sample first and repeated with the BASC Sample.

Sample size distributions by age for each model are presented in eTables 3–16 (Online Resource 1). Results were interpreted only across age ranges in which approximately 15 or more participants contributed observations, as estimates based on smaller samples may be unstable [19, 37]. For the CBCL sample, interpretations were limited to ages 4–18 in the intercept models; 6–17 for sex subgroups; 6–14 for NF1 inheritance subgroups; 7–14 for PN subgroups; and 7–11 for ADHD diagnosis subgroups. For the BASC sample, interpretations were limited to ages 3–18 in the intercept models; 3–15 for sex subgroups; 3–15 for NF1 inheritance mode subgroups; 9–13 for PN subgroups; and 8–15 for ADHD diagnosis subgroups.

## Results

### Sample characteristics

The CBCL Sample (*n* = 1,087) had a mean age of 10.94 years (*SD* = 3.61) with a range of 3–18 years. The BASC Sample (*n* = 754) had a mean age of 9.92 years (*SD* = 4.25) with a range of 3–18 years. Sample characteristics, including recruitment populations and data availability across sites, are presented in Tables 1 and 2, with additional details in eTables 1 and 2 (Online Resource 1). Descriptive statistics and correlations among study variables are presented in eTables 17, and 18 (Online Resource 1), with score ranges by age group shown in eTables 19, and 20 (Online Resource 1). In the CBCL sample, 23.6% of observations scored in the borderline or clinical range for internalizing problems, and 16.7% for externalizing problems; corresponding proportions in the BASC Sample were 31.1% and 23.9%, respectively. Substantial variability was observed across age groups for all focal study variables.

### Age trends of internalizing and externalizing problems in the total CBCL sample

**Fig. 2:**
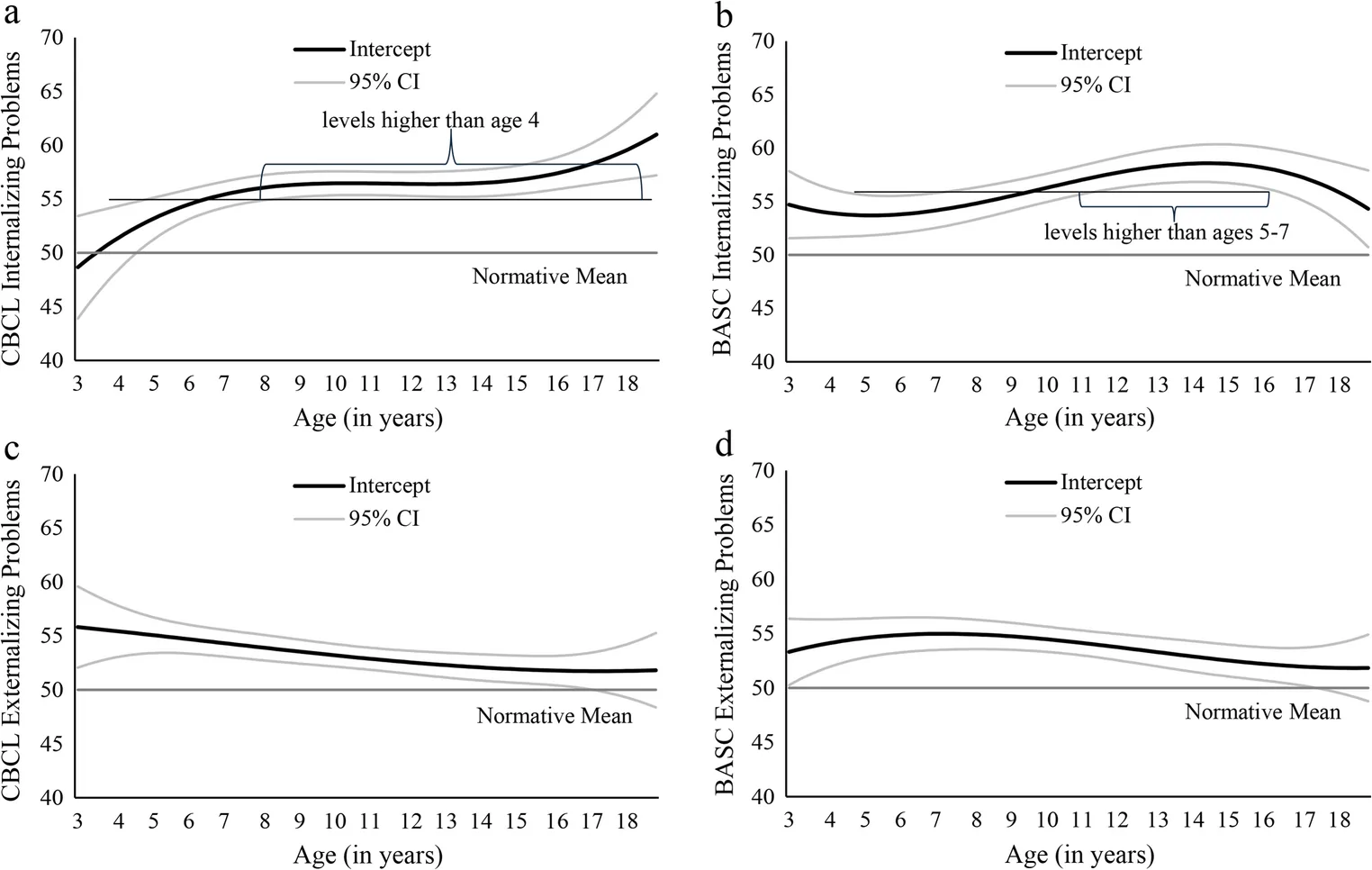
Age trends of internalizing and externalizing problems in total samples. Note. Outcomes included: Internalizing Problems by Child Behavior Checklist (CBCL) (**a**) and Behavior Assessment System for Children (BASC) (**b**), externalizing problems by CBCL (**c**) and BASC (**d**). Internalizing and externalizing problems are presented using standardized T scores (*M*_norm_ = 50, *SD*_norm_ = 10). Significant deviations from the normative sample are indicated by 95% confidence intervals (CIs) that do not include the normative mean of 50. Results for age ranges that had approximately 15 or more observations were interpreted

The age trends in parent-rated internalizing and externalizing problems in the CBCL sample are presented in Fig. 2 (see Online Resource 2: eTable 21 for coefficient estimates). The levels of internalizing problems were significantly higher than the normative mean from childhood to adolescence (ages 5 to 18), as indicated by 95% CIs that did not include the normative mean of 50 (Fig. 2a). This gap was significantly larger in mid-childhood to adolescence than in early childhood (e.g., non-overlapping 95% CIs at ages 4 vs. 8). Similarly, the levels of externalizing problems were significantly higher than the normative mean from childhood to adolescence (ages 4 to 17; Fig. 2c). However, the gap did not significantly vary across ages, despite the narrowing trend.

### Subgroup age trends of internalizing and externalizing problems in the CBCL sample

**Fig. 3:**
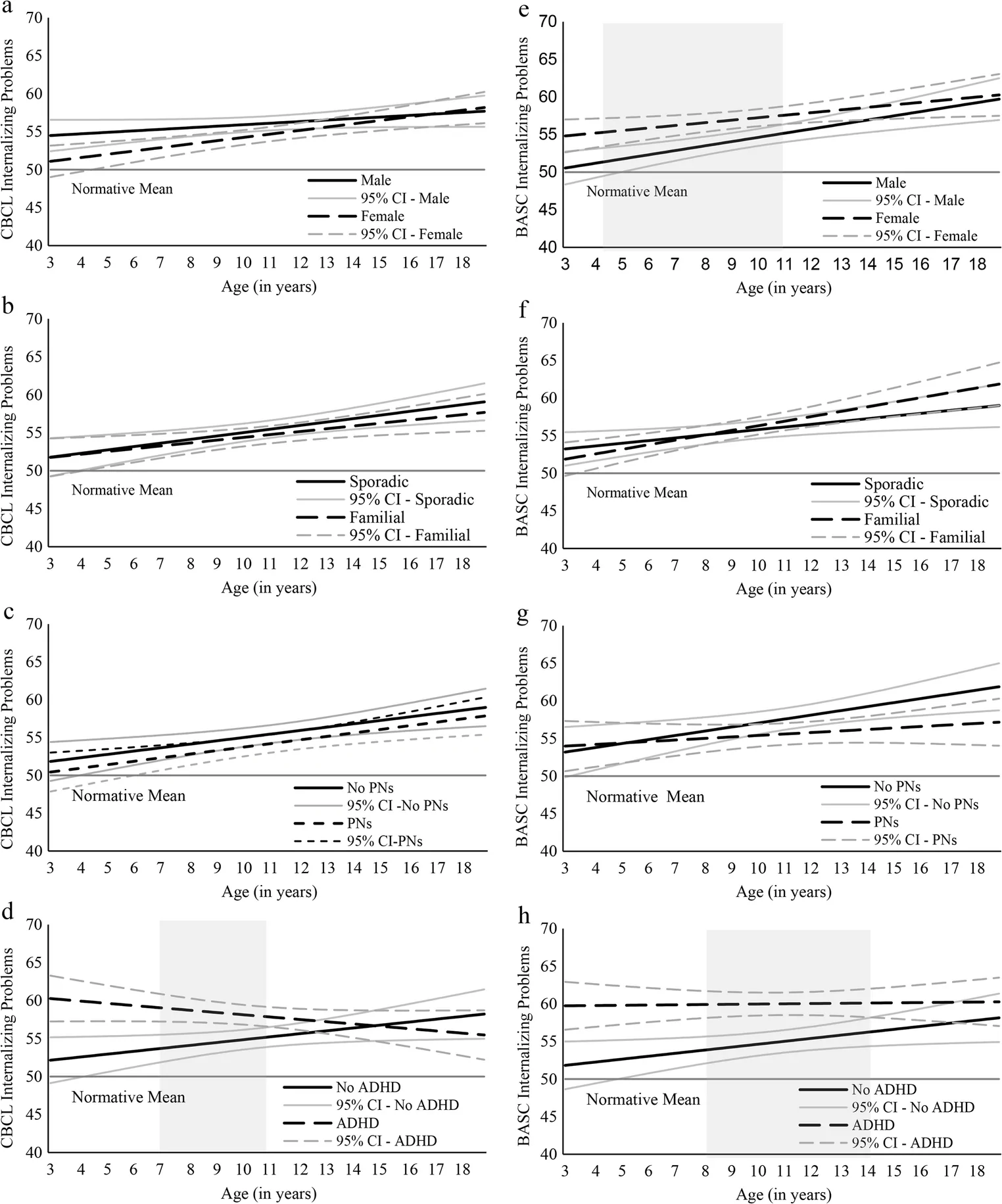
Age trends of internalizing problems in subgroups. *Note*. Figures present internalizing problems by Child Behavior Checklist (CBCL) across groups by Sex (**a**), NF1 Inheritance Mode (**b**), Plexiform Neurofibromas (PNs) (**c**), and ADHD Diagnosis (**d**); internalizing problems by Behavior Assessment System for Children (BASC) across groups by Sex (**e**), NF1 Inheritance Mode (**f**), PNs (**g**), and ADHD Diagnosis (**h**). Internalizing problems are presented using standardized T scores (*M*_norm_ = 50, *SD*_norm_ = 10). Significant deviations from the normative sample are indicated by 95% confidence intervals (CIs) that do not include the normative mean of 50. Significant group differences are indicated by non-overlapping 95% CIs between specific age points (grey areas). Results for age ranges that had approximately 15 or more observations were interpreted

**Fig. 4:**
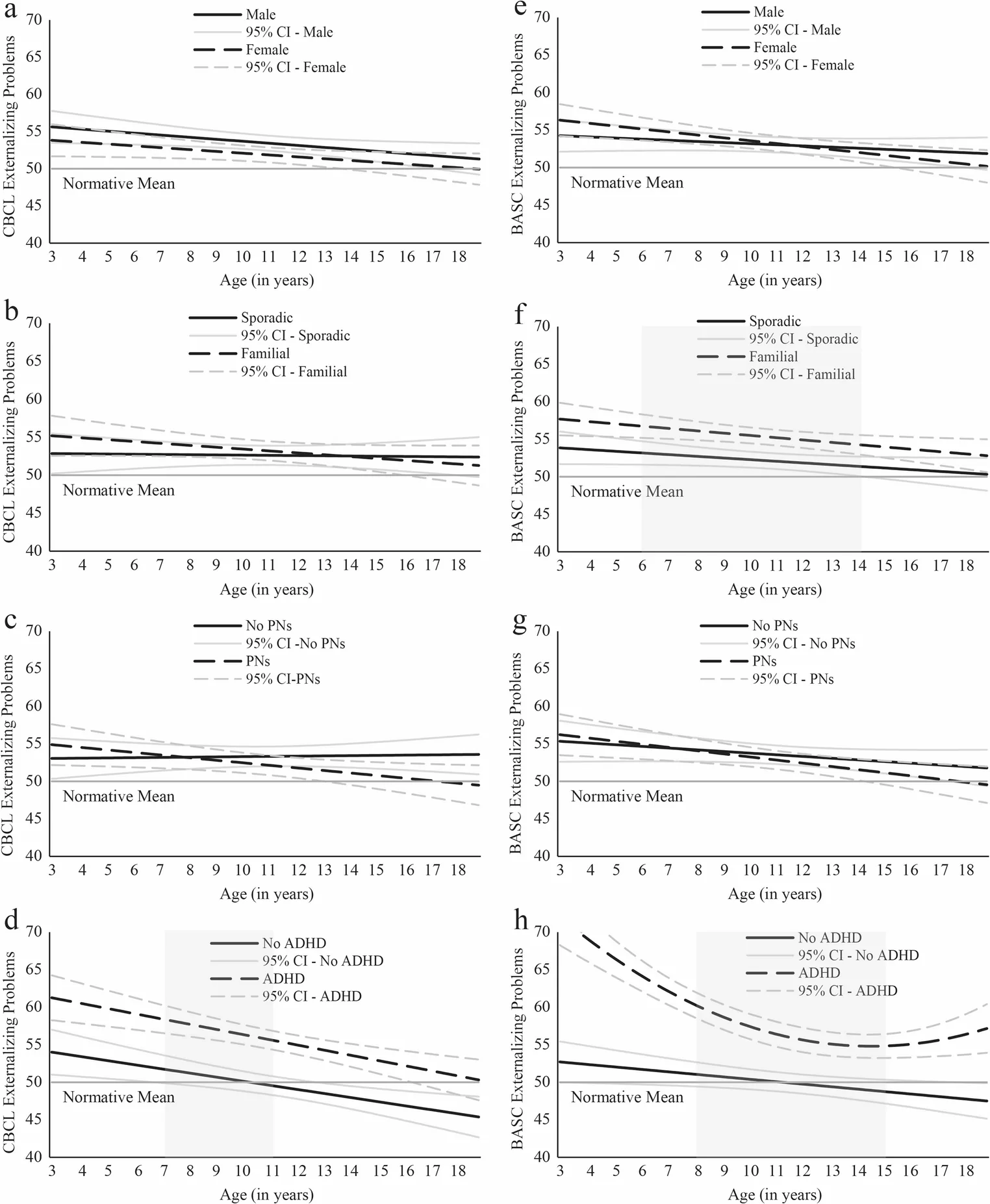
Age trends of externalizing problems in subgroups. Note. Figures present externalizing problems by Child Behavior Checklist (CBCL) across groups by Sex (**a**), NF1 Inheritance mode (**b**), Plexiform Neurofibromas (PNs) (**c**), and ADHD Diagnosis (**d**); internalizing problems by Behavior Assessment System for Children (BASC) across groups by Sex (**e**), NF1 Inheritance Mode (**f**), PNs (**g**), and ADHD Diagnosis (**h**). Externalizing problems are presented using standardized T scores (*M*_norm_ = 50, *SD*_norm_ = 10). Significant deviations from the normative sample are indicated by 95% confidence intervals (CIs) that do not include the normative mean of 50. Significant group differences are indicated by non-overlapping 95% CIs between specific age points (grey areas). Results for age ranges that had approximately 15 or more observations were interpreted

Across groups by sex, NF1 inheritance mode, and PNs status, levels and age patterns of internalizing problems were largely similar from early childhood to late adolescence, with no significant between-group differences at any ages, as indicated by overlapping 95% CIs (Figs. 3a–c; see coefficient estimates in Online Resource 2: eTables 22–24). Likewise, externalizing problems showed comparable levels and age patterns across these subgroups (Figs. 4a–c; see coefficient estimates in Online Resource 2: eTables 22–24).

In contrast, participants with ADHD exhibited significantly higher levels of both internalizing and externalizing problems compared to those without ADHD across substantial portions of childhood and adolescence (Figs. 3d and 4d; see coefficient estimates in Online Resource 2: eTable 25). Internalizing problems were greater among children with (vs. without) ADHD from ages 7 to 11. Externalizing problems were also higher in the ADHD group from ages 7 to 11, despite age-related declines in both groups; importantly, externalizing levels among children without ADHD were comparable to normative means, whereas elevations among those with ADHD remained evident, albeit with a narrowing gap with age.

### Results from replication analyses using the BASC sample

Age trends in internalizing and externalizing problems in the BASC sample largely replicated those observed in the CBCL sample, with minor differences in timing and subgroup effects (Figs. 2, 3 and 4). In the BASC sample, increases in internalizing problems were observed among older ages (approximately ages 8–14). Subgroup patterns were generally similar across measures, although sex differences in internalizing symptoms were slightly more apparent in the BASC sample. Externalizing problem patterns were highly consistent across measures, with small sample-specific differences, including modestly higher externalizing levels for familial versus sporadic NF1 in the BASC sample only. Coefficient estimates are provided in eTables 26–30 (Online Resource 2).

### Sensitivity analyses

Sensitivity analyses were conducted to test the age trends of children with NF1 across the internalizing and externalizing subdomains in both the CBCL and the BASC samples and across subgroups by sex, NF1 inheritance mode, PNs, and ADHD diagnosis. Results were largely consistent with those of the total problems and reported in the supplemental results (Online Resource 1).

Sensitivity analyses also examined potential site-related variability. Intraclass correlations indicated substantial clustering at the participant level (ICC = 0.50–0.77) but minimal clustering by site (ICC = 0.00005–0.04). We therefore compared TVEMs with participant-level random intercepts only to models additionally including site-level random intercepts, using likelihood ratio tests under maximum likelihood estimation. Including site-level random effects did not improve model fit for most models (Online Resource 1: eTables 31–32). Accordingly, primary results are reported from participant-level random-intercept models.

## Discussion

Existing psychosocial research on children with NF1 offers limited insight into age-related patterns of internalizing and externalizing problems and their predictors, which constrains the development of individualized, developmentally sensitive supports. Using two large, independent pooled samples assessed with widely used standardized measures, the current study helps address these gaps. Despite conceptual and structural differences between the CBCL and the BASC, age-related patterns of internalizing and externalizing problems were highly consistent across samples. This convergence strengthens confidence in the observed age patterns and supports their generalizability across measurement contexts.

Relative to normative data, children with NF1 exhibited significantly higher levels of parent-rated internalizing and externalizing problems from early childhood through middle adolescence. These findings extend prior work that documented elevated mean symptom levels pooled across ages by delineating age-specific patterns [14]. Although mean scores generally remained below clinical thresholds, they consistently exceeded population norms. Importantly, mean values masked substantial heterogeneity, with a sizeable proportion of children scoring in borderline or clinical ranges (17–31%). This elevation, even if subclinical on average, can confer vulnerability to impairments in daily functioning [39] and long-term developmental outcomes [40], underscoring the need for early identification, ongoing monitoring, and tailored supports for children and adolescents with NF1.

Internalizing problems showed greater deviation from normative expectations in older children and adolescents than in younger children. This pattern suggests that older youth with NF1 may face both typical developmental challenges and NF1-specific stressors that compound risk over time. Potential contributors include increasing disease burden related to physical features [41], progression of plexiform neurofibromas [23, 41], chronic pain [42], bullying and social exclusion [43], and cognitive or learning difficulties [44]. Social difficulties and autistic traits, which are common in NF1 [11] and associated with internalizing symptoms [45], may also become more salient during adolescence [46]. Sleep disturbances and heightened irritability—both frequently reported in NF1 [47, 48]—may further contribute to emotional dysregulation and parent-perceived internalizing symptoms. These interpretations should be viewed as hypothesis-generating and require confirmation in longitudinal, multi-informant studies.

In contrast, the elevation in parent-rated externalizing problems relative to norms remained relatively stable across ages. This pattern suggests that externalizing problems in children with NF1 may follow developmental trends similar to those observed in typically developing children, characterized by early increases and later stabilization [17]. However, NF1-related neurocognitive alterations—particularly in executive functioning [49] and inhibitory control [50] —may also influence these behaviors. Additional research is needed to determine whether the neurobiological mechanisms underlying externalizing problems in NF1 parallel or diverge from those observed in the general population.

Subgroup analyses showed that age patterns and magnitude of symptom elevations were largely consistent across sex, NF1 inheritance mode, and plexiform neurofibroma (PN) status, suggesting broadly shared vulnerability across these groups. These findings align with prior research [18, 21], although few studies have examined differences by PN status. Given that PNs may progress over time [23], longitudinal studies are needed to clarify whether PN changes relate to evolving symptom patterns. Minor discrepancies between samples also warrant further investigation. In contrast, children with comorbid ADHD exhibited substantially higher internalizing and externalizing problems across ages. These differences mirror findings from the general population [51] and may reflect shared mechanisms such as executive dysfunction [52, 53]. Given the elevated risk of ADHD [10] and executive function difficulties in NF1 [54], ADHD appears to be a key correlate of psychiatric burden. However, ADHD did not fully account for symptom elevations, especially in internalizing problems, as children without ADHD remained elevated relative to norms. Future research should assess ADHD dimensionally and examine neurobiological and family factors contributing to developmental variation in symptoms.

Several limitations should be considered. First, the cross-sectional design prevents assessment of within-individual changes over time. Future multi-center studies should prioritize longitudinal data collection across developmental periods. Second, all outcomes were based on parent reports, introducing potential mono-method and single-informant bias. Parents may underestimate internalizing symptoms and overestimate externalizing behaviors. Third, analyses relied on comparisons to normative data rather than unaffected controls assessed using parallel procedures. Although normative T scores provide clinically meaningful context, they cannot fully account for cohort or contextual effects; accordingly, age-related deviations should be interpreted as hypothesis-generating. Fourth, ADHD diagnostic status was based on parent report or clinical record, which were unavailable at several sites, limiting power for ADHD-related analyses and the age ranges with sufficient sample sizes for reliable estimates. In addition, substantial symptom overlap exists among ADHD, internalizing, and externalizing problems; future studies are needed to better characterize their comorbidity and developmental interplay. Fifth, subgroup analyses were constrained by limited sample sizes at certain ages; interpretation was therefore restricted to age ranges with adequate subgroup representation. Finally, data were drawn from community- and clinic-based samples assessed with two related but distinct instruments (CBCL and BASC). For instance, larger differences in BASC externalizing problems across ADHD diagnosis subgroups may reflect the measure’s inclusion of a hyperactivity subscale, a core ADHD symptom, unlike the CBCL. Although the replication of findings across measures supports the robustness of the identified age patterns, future well-designed, prospective longitudinal multicenter studies using consistent measures and standardized procedures are needed to more precisely characterize developmental trajectories.

## Conclusion

Using two large samples assessed with standardized measures, this study provides evidence that children and adolescents with NF1 experience age-related differences in internalizing and externalizing problems relative to population norms. Internalizing problems show greater deviations at older versus younger ages, whereas externalizing deviations are relatively similar across age. Although mean scores generally remained subclinical, substantial variability was observed, with a meaningful subset of children exhibiting borderline or clinical-level symptoms. Notably, comorbid ADHD emerged as a key correlate of both internalizing and externalizing problems across ages, particularly for externalizing symptoms, underscoring its relevance for risk stratification and clinical monitoring in NF1. While interpretation is tempered by reliance on parent report, predominantly cross-sectional data, and the absence of matched control groups, age-specific patterns highlight periods of vulnerability that may inform clinical surveillance and support. Overall, the findings underscore the need for longitudinal, multi-informant, and collaborative research to clarify developmental mechanisms, including the role of co-occurring ADHD, and to guide developmentally tailored, person-centered interventions for children with NF1.

## Supplementary Information

Below is the link to the electronic supplementary material.

Online Resource 1

Online Resource 2

## Data Availability

Main data generated in the current study are included in the manuscript and the supplementary documents. Additional data can be provided upon request to the corresponding author.
