# Supplementary material for: Age trends of internalizing and externalizing problems in children with neurofibromatosis type 1: a multicenter study
Source: Eur Child Adolesc Psychiatry. 2026 Apr 29;35(8):2621–32. doi: 10.1007/s00787-026-03039-x (PMC13652907; doi:10.1007/s00787-026-03039-x)
Supplement: Supplementary file 1 [file 787_2026_3039_MOESM3_ESM.pdf]

Supplementary Information

Age Trends of Internalizing and Externalizing Problems in Children with Neurofibromatosis Type 1: A Multicenter Study

European Journal of Child and Adolescent Psychiatry

Dan Liu[a], PhD, Xiaoli Zong[a,b], PhD, Jonathan M. Payne[c,d], PsyD, Peter L. Stavinoha[e], PhD, Pamela L. Wolters[f], PhD, Karin S. Walsh[g,h], PsyD, Jennifer Janusz[i], PsyD, Bonita P. Klein-Tasman[j], PhD, Stephanie M. Morris[k], MD, Carrie E. Bearden[l], PhD, Natalie Pride[m], PhD, Yang Hou[a], PhD

[a]Florida State University, Tallahassee, FL, USA

[b]Army Medical University, Chongqing, China

[c]Murdoch Children's Research Institute, Melbourne, Australia

[d]Department of Paediatrics, University of Melbourne, Melbourne, Australia

[e]University of Texas MD Anderson Cancer Center, Houston, TX, USA

[f]Pediatric Oncology Branch, Center for Cancer Research, National Cancer Institute, National Institutes of Health, Bethesda, MD, USA

[g]Children’s National Hospital, Washington, DC, USA

[h]The George Washington University School of Medicine, Washington, DC, USA

[i]Children’s Hospital Colorado, Aurora, CO, USA

[j]University of Wisconsin-Milwaukee, Milwaukee, WI, USA

[k]Kennedy Krieger Institute, Baltimore, MD, USA

[l]University of California, Los Angeles, CA, USA

[m]Children's Hospital at Westmead, Sydney, Australia

Correspondence to Yang Hou, Ph.D., Assistant Professor, Department of Behavioral Sciences and Social Medicine, College of Medicine, Florida State University, 1115 West Call Street, Tallahassee, Florida, 32306-4300, USA. Email: yang.hou@med.fsu.edu. Phone number:850-6456897

Table of Contents

Supplemental Descriptive Statistics 4
Supplemental Results of Internalizing and Externalizing Subdomains 5
eTable 1. Demographic, NF1 Disease-Related, and Clinical Characteristics Assessed at Each Site and in the Combined CBCL Sample (Sample 1) 7
eTable 2. Demographic, NF1 Disease-Related, and Clinical Characteristics Assessed at Each Site and in the Combined BASC Sample (Sample 2) 8
eTable 3. Sample Sizes for the Associations between Each Predictor and CBCL Internalizing Problems across Ages 9
eTable 4. Sample Sizes for the Associations between Each Predictor and CBCL Anxious/Depressed across Ages 10
eTable 5. Sample Sizes for the Associations between Each Predictor and CBCL Withdrawn/Depressed across Ages 11
eTable 6. Sample Sizes for the Associations between Each Predictor and CBCL Somatic Complaints across Ages 12
eTable 7. Sample Sizes for the Associations between Each Predictor and CBCL Externalizing Behaviors across Ages 13
eTable 8. Sample Sizes for the Associations between Each Predictor and CBCL Rulebreaking Behaviors across Ages 14
eTable 9. Sample Sizes for the Associations between Each Predictor and CBCL Aggressive Behaviors across Ages 15
eTable 10. Sample Sizes for the Associations between Each Predictor and BASC Internalizing Problems across Ages 16
eTable 11. Sample Sizes for the Associations between Each Predictor and BASC Anxiety across Ages 17
eTable 12. Sample Sizes for the Associations between Each Predictor and BASC Depression across Ages 18
eTable 13. Sample Sizes for the Associations between Each Predictor and BASC Somatization across Ages 19
eTable 14. Sample Sizes for the Associations between Each Predictor and BASC Externalizing Problems across Ages 20
eTable 15. Sample Sizes for the Associations between Each Predictor and BASC Conduct Problems across Ages 21
eTable 16. Sample Sizes for the Associations between Each Predictor and BASC Aggression across Ages 22
eTable 17. Descriptives and Correlations of CBCL Outcomes and Correlates 23
eTable 18. Descriptives and Correlations of BASC Outcomes and Correlates 24
eTable 19. Score Range by Age in the CBCL Sample 25
eTable 20. Score Range by Age in the BASC Sample 26
eTable 21. TVEM Estimates for CBCL Internalizing and Externalizing Problems in the Overall Sample (see Online Resource 2) 27
eTable 22. TVEM Estimates for CBCL Internalizing and Externalizing Problems across Sex Groups (see Online Resource 2) 27
eTable 23. TVEM Estimates for CBCL Internalizing and Externalizing Problems across NF1 Inheritance Mode Groups (see Online Resource 2) 27
eTable 24. TVEM Estimates for CBCL Internalizing and Externalizing Problems across Plexiform Neurofibromas Groups (see Online Resource 2) 27
eTable 25. TVEM Estimates for CBCL Internalizing and Externalizing Problems across ADHD Diagnosis Groups (see Online Resource 2) 27
eTable 26. TVEM Estimates for BASC Internalizing and Externalizing Problems in the Overall Sample (see Online Resource 2) 27
eTable 27. TVEM Estimates for BASC Internalizing and Externalizing Problems across Sex Groups (see Online Resource 2) 27
eTable 28. TVEM Estimates for BASC Internalizing and Externalizing Problems across NF1 Inheritance Mode Groups (see Online Resource 2) 27

eTable 29. TVEM Estimates for BASC Internalizing and Externalizing Problems across Plexiform Neurofibromas Groups (see Online Resource 2) ... 27
eTable 30. TVEM Estimates for BASC Internalizing and Externalizing Problems across ADHD Diagnosis Groups (see Online Resource 2) ... 27
eTable 31. Model Fit Comparison Across Random Effect Specifications in the CBCL Sample ... 28
eTable 32. Model Fit Comparison Across Random Effect Specifications in the BASC Sample ... 35
eFigure 1. Age Trends of Subdomains of Internalizing Problems ... 41
eFigure 2. Age Trends of Subdomains of Externalizing Problems ... 43
eFigure 3. Age Trends of Subdomains of Internalizing Problems across Sex Groups ... 44
eFigure 4. Age Trends of Subdomains of Externalizing Problems across Sex Groups ... 46
eFigure 5. Age Trends of Subdomains of Internalizing Problems across NF1 Inheritance Mode Groups ... 47
eFigure 6. Age Trends of Subdomains of Externalizing Problems across NF1 Inheritance Mode Groups ... 49
eFigure 7. Age Trends of Subdomains of Internalizing Problems across Plexiform Neurofibromas Groups ... 50
eFigure 8. Age Trends of Subdomains of Externalizing Problems across Plexiform Neurofibromas Groups ... 52
eFigure 9. Age Trends of Subdomains of Internalizing Problems across ADHD Diagnosis Groups ... 53
eFigure 10. Age Trends of Subdomains of Externalizing Problems across ADHD Diagnosis Groups ... 55

Supplemental Descriptive Statistics

eTables 17-18 present the descriptives and correlations of all study variables in the CBCL sample (sample assessed with the Child Behavior Checklist) and in the BASC sample (sample assessed with Behavior Assessment System for Children), respectively. The means of total internalizing and total externalizing problems as well as all subdomains in children with NF1 were higher than the normative mean of 50 in both samples. Minimum mean differences were observed for conduct problems and aggression in the BASC sample. Internalizing problems (total and subdomains) were strongly and positively correlated with each other and weakly to strongly and positively correlated with externalizing problems (total and subdomains). Total and subdomains of externalizing problems were also strongly and positively correlated with each other. Older children generally had higher levels of internalizing problems and lower levels of externalizing problems (total and subdomains) than the normative sample. Correlations between sex and internalizing/externalizing problems were mostly weak and somewhat inconsistent across the two samples. NF1 inheritance mode was weakly and positively correlated with depression and externalizing problems (total and subdomains) in the BASC sample only. The presence of plexiform neurofibromas (PNs) was only weakly and negatively correlated with anxiety problems in the BASC sample. ADHD diagnosis status was generally positively correlated with total and subdomains of internalizing and externalizing problems in both samples, with the magnitude being weak to moderate.

Supplemental Results of Internalizing and Externalizing Subdomains

Initial Analysis with the CBCL Sample

Age-related patterns for internalizing subdomains—including anxious/depressed (eFigure 1A1), withdrawn/depressed (eFigure 1B1), and somatic complaints (eFigure 1C1)—were broadly similar to those observed for total internalizing problems. Across ages, scores showed modest elevations from early childhood to middle adolescence, with minor differences in the shapes of the age-related curves. Differences between participants and the normative sample in these internalizing subdomains were also generally consistent with those observed for total internalizing problems (Figure 1A). Somatic complaints appeared to be slightly elevated relative to total internalizing problems across much of the age span.

For externalizing subdomains, rule-breaking behaviors showed relatively little age-related variation from early childhood to late adolescence (ages 4–18; eFigure 2A1), whereas aggressive behaviors displayed modestly lower levels (not statistically significant) at older compared to younger ages (eFigure 2B1), consistent with patterns observed for total externalizing problems (Figure 1C). Both rule-breaking and aggressive behaviors appeared somewhat elevated relative to total externalizing problems.

Age-related patterns in internalizing subdomains were largely comparable across sex (eFigure 3), NF1 inheritance mode (eFigure 5), PN status (eFigure 7), and ADHD diagnosis (eFigure 9) groups, with several small differences. Males exhibited slightly higher withdrawn/depressed scores than females between ages 6 and 11. Participants without (versus with) PNs showed somewhat higher somatic complaints between ages 10 and 12. The age trend of somatic complains across PNs groups also differed, with the no-PNs group being linear across ages and the PNs group being U-shaped (lowest scores between ages 9-12). Participants with ADHD had slightly but significantly higher anxious/depressed (ages 8–11) and withdrawn/depressed scores (ages 7–10) relative to those without ADHD. Somatic complaints displayed differing age-related patterns by ADHD diagnosis status.

Externalizing subdomains showed few subgroup differences by sex (eFigure 4), NF1 inheritance mode (eFigure 6), or PN status (eFigure 8). ADHD diagnosis status was associated with higher rule-breaking behaviors (ages 9–13) and aggressive behaviors (ages 7–13) relative to participants without ADHD (eFigure 10).

Replication Analysis Using the BASC Sample

In the BASC sample, age-related patterns in anxiety, depression, and somatization (eFigures 1A2–1C2) were generally consistent with total internalizing problems. Scores were relatively similar in early childhood (ages 3–8), were highest around mid-adolescence (approximately age 15), and were slightly lower (not statistically significant) at older ages. Compared to anxious/depressed in the CBCL sample, BASC anxiety demonstrated greater variation across ages, characterized by early levels near the normative mean followed by higher scores in mid-adolescence and lower (not statistically significant) scores at older ages. Differences between withdrawn/depressed (CBCL) and depression (BASC) paralleled overall differences observed for total internalizing problems across the two samples. Somatization scores in the BASC sample appeared lower across ages compared to CBCL somatic complaints.

For externalizing subdomains, conduct problems and aggression (eFigures 2A2–2B2) appeared to be lower across ages relative to rule-breaking and aggressive behaviors in the CBCL sample and also appeared to be lower than total externalizing problems within the BASC sample. Deviations from normative means were minimal.

Subgroup analyses in the BASC sample revealed several distinctions relative to the CBCL findings. Across sex groups (eFigures 3–4), females showed higher anxiety than males between ages 5 and 9; the age trends also differed. Depression levels or age trend did not differ by sex. Somatization levels were slightly but significantly higher among females around age 8. Conduct problems appeared to be lower among males compared to scores of rulebreaking behaviors in the CBCL sample, while females exhibited lower levels at older ages.

Across NF1 inheritance mode groups (eFigures 5–6), anxiety showed stronger age-related elevations in both groups

relative to the CBCL sample. Somatization was significantly higher at older ages among participants with familial NF1 while it did not vary across ages for children with sporadic NF1. Levels of conduct problems differed at specific ages (5–7 and 13–14), with participants with familial NF1 showing higher scores than those with sporadic NF1 in the BASC sample; the two groups also differed in age-related trend. Aggression was slightly but significantly higher among participants with familial NF1 between ages 8 and 10.

Across PN status groups (eFigures 7–8), anxiety was higher at ages 10–13 among participants without PNs in the BASC sample. Conduct problems were significantly lower at older ages across both groups in the BASC sample. Age-related patterns of aggression levels differed by PN status.

Across ADHD groups (eFigures 9–10), anxiety and depression were higher between ages 9 and 11 among participants with ADHD compared to those without ADHD. Somatization did not differ substantially by ADHD diagnosis status. ADHD-related group differences in conduct problems and aggression appeared to be more pronounced in the BASC sample than in the CBCL sample, with the no-ADHD group showing levels the same as the normative sample. In general, the age-related patterns of group differences by ADHD diagnosis status in internalizing and externalizing subdomains were consistent across the CBCL and BASC samples.

eTable 1. Demographic, NF1 Disease-Related, and Clinical Characteristics Assessed at Each Site and in the Combined CBCL Sample (Sample 1)

**Characteristics**	**University of Texas, MD Anderson Cancer Center, USA**	**Children's National Hospital, USA**	**University of California, Los Angeles, USA**	**Murdoch Children's Research Institute, Australia**	**Children's Hospital at Westmead, Australia**	**Children's Hospital Colorado, USA**	**Combined**
Informant	Medical records	Demographic information was reported by parents or primary caregivers; NF1-related disease characteristics were rated by medical professionals	Reported by parents or primary caregivers	Reported by parents or primary caregivers	Reported by parents or primary caregivers	Medical records	NA
Child sex	1 = Male, 2 = Female	1 = Male, 2 = Female, 3 = Gender nonconforming or transgender	1 = Male, 2 = Female	1 = Male, 2 = Female	1 = Male, 2 = Female	0 = Female, 1 = Male	0 = Male, 1= Female
NF1 inheritance mode	Verified diagnosis of NF1 in patient's father/mother: 0 = No, 1 = Yes	NA	NA	1 = Sporadic, 2 = Familial	1 = Sporadic, 2 = Familial	NF1 status of father/mother: 1 = Affected, 2 = No	0 = Sporadic (neither parent had NF1), 1 = Familial (at least one parent had NF1)
Plexiform neurofibromas	0 = Not noted in medical record, 1 = Yes	NA	NA	1 = Yes, 2 = No	1 = Yes, 2 = No	0 = Absent, 1 = Present	0 = no; 1 = yes
ADHD diagnosis	NA	Check if neurodevelopmental disorders diagnosis/diagnoses = ADHD	Extracted from clinical information	Existing diagnosis prior to our assessment = ADHD	Existing diagnosis prior to our assessment = ADHD	1=YES; 0=NO	0 = no; 1 = yes

Note. CBCL = Child Behavior Checklist. NF1 = neurofibromatosis type 1. ADHD = attention-deficit/hyperactivity disorder. NA = not applicable.

eTable 2. Demographic, NF1 Disease-Related, and Clinical Characteristics Assessed at Each Site and in the Combined BASC Sample (Sample 2)

**Characteristics**	**National Cancer Institute, USA**	**Children's Hospital Colorado, USA**	**University of Wisconsin–Milwaukee, USA**	**Washington University in St. Louis, USA**	**Children's Hospital at Westmead, Australia**	**University of Texas, MD Anderson Cancer Center, USA**	**Combined**
Informant	Demographic information and NF1 Inheritance mode were reported by parents or primary caregivers; other variables were rated by medical professionals	Medical records	Reported by parents or primary caregivers	Medical records	Reported by parents or primary caregivers	Medical records	NA
Child sex	0 = Female, 1 = Male	0 = Female, 1 = Male	1 = Male, 2 = Female	0 = Male, 1= Female	1 = Male, 2 = Female	1 = Male, 2 = Female	0 = Male, 1= Female
NF1 inheritance mode	NF1 status of father/mother: 1 = Affected, 2 = No	NF1 status of father/mother: 1 = Affected, 2 = No	1 = Familial, 2 = Sporadic	Family History of NF1: 0 = No, 1 = Yes	1 = Sporadic, 2 = Familial	Verified diagnosis of NF1 in patient's father/mother: 0 = No, 1 = Yes	0 = Sporadic (neither parent had NF1), 1 = Familial (at least one parent had NF1)
Plexiform neurofibromas	0 = Absent, 1 = Present	0 = Absent, 1 = Present	TRUE = Yes, FALSE = No	NA	1 = Yes, 2 = No	0 = Not noted in medical record, 1 = Yes	0 = no; 1 = yes
ADHD diagnosis	1=YES; 0=NO	1=YES; 0=NO	NA	NA	Existing diagnosis prior to our assessment = ADHD	NA	0 = no; 1 = yes

Note. BASC = Behavior Assessment System for Children. NF1 = neurofibromatosis type 1. ADHD = attention-deficit/hyperactivity disorder. NA = not applicable.

eTable 3. Sample Sizes for the Associations between Each Predictor and CBCL Internalizing Problems across Ages

Predictor	Age Group (in years)															
	**3.00-3.99**	**4.00-4.99**	**5.00-5.99**	**6.00-6.99**	**7.00-7.99**	**8.00-8.99**	**9.00-9.99**	**10.00-10.99**	**11.00-11.99**	**12.00-12.99**	**13.00-13.99**	**14.00-14.99**	**15.00-15.99**	**16.00-16.99**	**17.00-17.99**	**18.00-18.99**
Intercept	9	29	52	70	91	86	107	92	95	92	82	79	67	47	38	18
Sex	9	29	51	69	91	86	106	92	95	92	81	78	67	47	38	18
Male	7	16	38	40	34	45	55	55	50	45	45	40	38	22	19	11
Female	2	13	13	29	57	41	51	37	45	47	36	38	29	25	19	7
NF1 inheritance mode	5	23	39	52	68	55	74	66	67	74	61	52	44	35	28	9
Sporadic	2	16	28	30	45	30	39	36	40	48	35	30	31	22	16	5
Familial	3	7	11	22	23	25	35	30	27	26	26	22	13	13	12	4
Plexiform neurofibromas	4	24	40	47	60	52	69	59	65	71	60	52	46	35	30	12
No	4	14	29	35	43	41	52	43	42	50	39	30	32	25	18	7
Yes	10	10	11	12	17	11	17	16	23	21	21	22	14	10	12	5
ADHD diagnosis	6	8	15	28	37	38	47	39	38	28	31	29	26	13	9	8
No	6	5	4	17	21	19	25	21	21	17	17	17	17	7	6	2
Yes	0	3	11	11	16	19	22	18	17	11	14	12	9	6	3	6

Note. CBCL = Child Behavior Checklist. NF1 = neurofibromatosis type 1. ADHD = attention-deficit/hyperactivity disorder.

eTable 4. Sample Sizes for the Associations between Each Predictor and CBCL Anxious/Depressed across Ages

Predictor	Age Group (in years)															
	**3.00-3.99**	**4.00-4.99**	**5.00-5.99**	**6.00-6.99**	**7.00-7.99**	**8.00-8.99**	**9.00-9.99**	**10.00-10.99**	**11.00-11.99**	**12.00-12.99**	**13.00-13.99**	**14.00-14.99**	**15.00-15.99**	**16.00-16.99**	**17.00-17.99**	**18.00-18.99**
Intercept	9	29	57	72	99	92	108	92	100	94	84	79	67	48	38	18
Sex	9	29	56	71	99	92	107	92	100	94	83	78	67	48	38	18
Male	7	16	42	42	38	48	56	55	54	45	46	40	37	23	19	11
Female	2	13	14	29	61	44	51	37	46	49	37	38	30	25	19	7
NF1 inheritance mode	5	23	42	52	68	55	74	66	67	75	61	52	44	35	28	9
Sporadic	2	16	29	30	45	30	39	36	40	49	35	30	31	22	16	5
Familial	3	7	13	22	23	25	35	30	27	26	26	22	13	13	12	4
Plexiform neurofibromas	3	21	40	47	60	52	69	59	65	71	60	52	46	35	30	12
No	3	11	28	35	43	41	52	43	42	50	39	30	32	25	18	7
Yes	0	10	12	12	17	11	17	16	23	21	21	22	14	10	12	5
ADHD diagnosis	2	4	8	17	30	43	44	48	39	43	29	33	29	27	14	9
No	2	4	5	4	17	25	21	25	21	24	17	18	17	18	8	6
Yes	0	3	13	13	18	23	23	18	19	12	15	12	9	6	3	6

Note. CBCL = Child Behavior Checklist. NF1 = neurofibromatosis type 1. ADHD = attention-deficit/hyperactivity disorder.

eTable 5. Sample Sizes for the Associations between Each Predictor and CBCL Withdrawn/Depressed across Ages

Predictor	Age Group (in years)															
	**3.00-3.99**	**4.00-4.99**	**5.00-5.99**	**6.00-6.99**	**7.00-7.99**	**8.00-8.99**	**9.00-9.99**	**10.00-10.99**	**11.00-11.99**	**12.00-12.99**	**13.00-13.99**	**14.00-14.99**	**15.00-15.99**	**16.00-16.99**	**17.00-17.99**	**18.00-18.99**
Intercept	2	16	23	71	99	92	108	92	100	94	83	79	68	48	38	18
Sex	2	16	23	70	99	92	107	92	100	94	82	78	68	48	38	18
Male	0	9	15	41	38	48	56	55	54	45	46	40	38	23	19	11
Female	2	7	8	29	61	44	51	37	46	49	36	38	30	25	19	7
NF1 inheritance mode	2	16	23	52	68	55	74	66	67	75	61	52	44	35	28	9
Sporadic	1	10	18	30	45	30	39	36	40	49	35	30	31	22	16	5
Familial	1	6	5	22	23	25	35	30	27	26	26	22	13	13	12	4
Plexiform neurofibromas	1	16	23	47	60	52	69	59	65	71	60	52	46	35	30	12
No	1	9	17	35	43	41	52	43	42	50	39	30	32	25	18	7
Yes	0	7	6	12	17	11	17	16	23	21	21	22	14	10	12	5
ADHD diagnosis	1	0	0	29	43	44	48	39	43	29	32	29	27	14	9	8
No	1	0	0	16	25	21	25	21	24	17	17	17	18	8	6	2
Yes	0	0	0	13	18	23	23	18	19	12	15	12	9	6	3	6

Note. CBCL = Child Behavior Checklist. NF1 = neurofibromatosis type 1. ADHD = attention-deficit/hyperactivity disorder.

eTable 6. Sample Sizes for the Associations between Each Predictor and CBCL Somatic Complaints across Ages

Predictor	Age Group (in years)															
	**3.00-3.99**	**4.00-4.99**	**5.00-5.99**	**6.00-6.99**	**7.00-7.99**	**8.00-8.99**	**9.00-9.99**	**10.00-10.99**	**11.00-11.99**	**12.00-12.99**	**13.00-13.99**	**14.00-14.99**	**15.00-15.99**	**16.00-16.99**	**17.00-17.99**	**18.00-18.99**
Intercept	9	29	57	72	99	91	108	92	100	94	83	79	68	48	38	18
Sex	9	29	56	71	99	91	107	92	100	94	82	78	68	48	38	18
Male	7	16	42	42	38	47	56	55	54	45	46	40	38	23	19	11
Female	2	13	14	29	61	44	51	37	46	49	36	38	30	25	19	7
NF1 inheritance mode	5	23	42	52	68	54	74	66	67	75	61	52	44	35	28	9
Sporadic	2	16	29	30	45	29	39	36	40	49	35	30	31	22	16	5
Familial	3	7	13	22	23	25	35	30	27	26	26	22	13	13	12	4
Plexiform neurofibromas	4	24	43	47	60	51	69	59	65	71	60	52	46	35	30	12
No	4	14	31	35	43	40	52	43	42	50	39	30	32	25	18	7
Yes	0	10	12	12	17	11	17	16	23	21	21	22	14	10	12	5
ADHD diagnosis	6	8	17	30	43	43	48	39	43	29	32	29	27	14	9	8
No	6	5	4	17	25	21	25	21	24	17	17	17	18	8	6	2
Yes	0	3	13	13	18	22	23	18	19	12	15	12	9	6	3	6

Note. CBCL = Child Behavior Checklist. NF1 = neurofibromatosis type 1. ADHD = attention-deficit/hyperactivity disorder.

eTable 7. Sample Sizes for the Associations between Each Predictor and CBCL Externalizing Behaviors across Ages

**Predictor**	**Age Group (in years)**															
	**3.00-3.99**	**4.00-4.99**	**5.00-5.99**	**6.00-6.99**	**7.00-7.99**	**8.00-8.99**	**9.00-9.99**	**10.00-10.99**	**11.00-11.99**	**12.00-12.99**	**13.00-13.99**	**14.00-14.99**	**15.00-15.99**	**16.00-16.99**	**17.00-17.99**	**18.00-18.99**
Intercept	9	29	55	70	93	86	107	92	95	92	82	79	67	47	38	18
Sex	9	29	54	69	93	86	106	92	95	92	81	78	67	47	38	18
Male	7	16	40	40	35	45	55	55	50	45	45	40	38	22	19	11
Female	2	13	14	29	58	41	51	37	45	47	36	38	29	25	19	7
NF1 inheritance mode	5	23	42	52	68	55	74	66	67	74	61	52	44	35	28	9
Sporadic	2	16	29	30	45	30	39	36	40	48	35	30	31	22	16	5
Familial	3	7	13	22	23	25	35	30	27	26	26	22	13	13	12	4
Plexiform neurofibromas	4	24	43	47	60	52	69	59	65	71	60	52	46	35	30	12
No	4	14	31	35	43	41	52	43	42	50	39	30	32	25	18	7
Yes	0	10	12	12	17	11	17	16	23	21	21	22	14	10	12	5
ADHD diagnosis	6	8	15	28	37	38	47	39	38	28	31	29	26	13	9	8
No	6	5	4	17	21	19	25	21	21	17	17	17	17	7	6	2
Yes	0	3	11	11	16	19	22	18	17	11	14	12	9	6	3	6

Note. CBCL = Child Behavior Checklist. NF1 = neurofibromatosis type 1. ADHD = attention-deficit/hyperactivity disorder.

eTable 8. Sample Sizes for the Associations between Each Predictor and CBCL Rulebreaking Behaviors across Ages

Predictor	Age Group (in years)															
	**3.00-3.99**	**4.00-4.99**	**5.00-5.99**	**6.00-6.99**	**7.00-7.99**	**8.00-8.99**	**9.00-9.99**	**10.00-10.99**	**11.00-11.99**	**12.00-12.99**	**13.00-13.99**	**14.00-14.99**	**15.00-15.99**	**16.00-16.99**	**17.00-17.99**	**18.00-18.99**
Intercept	1	16	23	71	99	92	108	92	100	93	83	79	68	48	38	18
Sex	1	16	23	70	99	92	107	92	100	93	82	78	68	48	38	18
Male	0	9	15	41	38	48	56	55	54	45	46	40	38	23	19	11
Female	1	7	8	29	61	44	51	37	46	48	36	38	30	25	19	7
NF1 inheritance mode	1	16	23	52	68	55	74	66	67	74	61	52	44	35	28	9
Sporadic	1	10	18	30	45	30	39	36	40	49	35	30	31	22	16	5
Familial	0	6	5	22	23	25	35	30	27	25	26	22	13	13	12	4
Plexiform neurofibromas	1	16	23	47	60	52	69	59	65	70	60	52	46	35	30	12
No	1	9	17	35	43	41	52	43	42	49	39	30	32	25	18	7
Yes	0	7	6	12	17	11	17	16	23	21	21	22	14	10	12	5
ADHD diagnosis	0	0	0	29	43	44	48	39	43	29	32	29	27	14	9	8
No	0	0	0	16	25	21	25	21	24	17	17	17	18	8	6	2
Yes	0	0	0	13	18	23	23	18	19	12	15	12	9	6	3	6

Note. CBCL = Child Behavior Checklist. NF1 = neurofibromatosis type 1. ADHD = attention-deficit/hyperactivity disorder.

eTable 9. Sample Sizes for the Associations between Each Predictor and CBCL Aggressive Behaviors across Ages

**Predictor**	**Age Group (in years)**															
	**3.00-3.99**	**4.00-4.99**	**5.00-5.99**	**6.00-6.99**	**7.00-7.99**	**8.00-8.99**	**9.00-9.99**	**10.00-10.99**	**11.00-11.99**	**12.00-12.99**	**13.00-13.99**	**14.00-14.99**	**15.00-15.99**	**16.00-16.99**	**17.00-17.99**	**18.00-18.99**
Intercept	8	26	54	72	99	92	108	92	100	94	83	79	68	48	38	18
Sex	8	26	53	71	99	92	107	92	100	94	82	78	68	48	38	18
Male	6	14	40	42	38	48	56	55	54	45	46	40	38	23	19	11
Female	2	12	13	29	61	44	51	37	46	49	36	38	30	25	19	7
NF1 inheritance mode	4	20	39	52	68	55	74	66	67	75	61	52	44	35	28	9
Sporadic	1	14	26	30	45	30	39	36	40	49	35	30	31	22	16	5
Familial	3	6	13	22	23	25	35	30	27	26	26	22	13	13	12	4
Plexiform neurofibromas	3	21	40	47	60	52	69	59	65	71	60	52	46	35	30	12
No	3	11	28	35	43	41	52	43	42	50	39	30	32	25	18	7
Yes	0	10	12	12	17	11	17	16	23	21	21	22	14	10	12	5
ADHD diagnosis	5	5	14	30	43	44	48	39	43	29	32	29	27	14	9	8
No	5	2	3	17	25	21	25	21	24	17	17	17	18	8	6	2
Yes	0	3	11	13	18	23	23	18	19	12	15	12	9	6	3	6

Note. CBCL = Child Behavior Checklist. NF1 = neurofibromatosis type 1. ADHD = attention-deficit/hyperactivity disorder.

eTable 10. Sample Sizes for the Associations between Each Predictor and BASC Internalizing Problems across Ages

**Predictor**	**Age Group (in years)**															
	**3.00-3.99**	**4.00-4.99**	**5.00-5.99**	**6.00-6.99**	**7.00-7.99**	**8.00-8.99**	**9.00-9.99**	**10.00-10.99**	**11.00-11.99**	**12.00-12.99**	**13.00-13.99**	**14.00-14.99**	**15.00-15.99**	**16.00-16.99**	**17.00-17.99**	**18.00-18.99**
Intercept	47	58	67	70	44	58	53	60	46	45	50	40	36	33	30	17
Sex	47	58	67	70	44	58	53	60	46	45	50	40	36	33	30	17
Male	28	34	38	37	17	31	31	31	24	32	25	21	19	19	18	10
Female	19	24	29	33	27	27	22	29	22	13	25	19	17	14	12	7
NF1 inheritance mode	45	58	65	69	43	53	50	56	44	41	43	39	35	33	28	16
Sporadic	28	35	43	37	26	36	38	38	28	24	30	22	18	20	17	11
Familial	17	23	22	32	17	17	12	18	16	17	13	17	17	13	11	5
Plexiform neurofibromas	21	23	24	28	20	26	44	41	34	33	42	34	27	25	27	16
No	11	13	13	13	10	15	21	19	20	15	17	14	9	8	6	4
Yes	10	10	11	15	10	11	23	22	14	18	25	20	18	17	21	12
ADHD diagnosis	25	27	29	27	23	30	33	36	36	32	37	35	30	29	29	17
No	24	24	20	19	10	12	19	19	18	23	24	18	14	18	20	13
Yes	1	3	9	8	13	18	14	17	18	9	13	17	16	11	9	4

Note. BASC = Behavior Assessment System for Children. NF1 = neurofibromatosis type 1. ADHD = attention-deficit/hyperactivity disorder.

eTable 11. Sample Sizes for the Associations between Each Predictor and BASC Anxiety across Ages

**Predictor**	**Age Group (in years)**															
	**3.00-3.99**	**4.00-4.99**	**5.00-5.99**	**6.00-6.99**	**7.00-7.99**	**8.00-8.99**	**9.00-9.99**	**10.00-10.99**	**11.00-11.99**	**12.00-12.99**	**13.00-13.99**	**14.00-14.99**	**15.00-15.99**	**16.00-16.99**	**17.00-17.99**	**18.00-18.99**
Intercept	47	58	67	70	44	57	53	60	46	45	50	39	35	33	30	17
Sex	47	58	67	70	44	57	53	60	46	45	50	39	35	33	30	17
Male	28	34	38	37	17	30	31	31	24	32	25	20	19	19	18	10
Female	19	24	29	33	27	27	22	29	22	13	25	19	16	14	12	7
NF1 inheritance mode	45	58	65	69	43	52	50	56	44	41	43	38	34	33	28	16
Sporadic	28	35	43	37	26	35	38	38	28	24	30	21	17	20	17	11
Familial	17	23	22	32	17	17	12	18	16	17	13	17	17	13	11	5
Plexiform neurofibromas	21	23	24	28	20	25	44	41	34	33	42	33	26	25	27	16
No	11	13	13	13	10	14	21	19	20	15	17	13	8	8	6	4
Yes	10	10	11	15	10	11	23	22	14	18	25	20	18	17	21	12
ADHD diagnosis	25	27	29	27	23	29	33	36	36	32	37	34	29	29	29	17
No	24	24	20	19	10	11	19	19	18	23	24	17	13	18	20	13
Yes	1	3	9	8	13	18	14	17	18	9	13	17	16	11	9	4

Note. BASC = Behavior Assessment System for Children. NF1 = neurofibromatosis type 1. ADHD = attention-deficit/hyperactivity disorder.

eTable 12. Sample Sizes for the Associations between Each Predictor and BASC Depression across Ages

**Predictor**	**Age Group (in years)**															
	**3.00-3.99**	**4.00-4.99**	**5.00-5.99**	**6.00-6.99**	**7.00-7.99**	**8.00-8.99**	**9.00-9.99**	**10.00-10.99**	**11.00-11.99**	**12.00-12.99**	**13.00-13.99**	**14.00-14.99**	**15.00-15.99**	**16.00-16.99**	**17.00-17.99**	**18.00-18.99**
Intercept	47	58	67	70	44	57	53	60	46	45	50	39	35	33	30	17
Sex	47	58	67	70	44	57	53	60	46	45	50	39	35	33	30	17
Male	28	34	38	37	17	30	31	31	24	32	25	20	19	19	18	10
Female	19	24	29	33	27	27	22	29	22	13	25	19	16	14	12	7
NF1 inheritance mode	45	58	65	69	43	52	50	56	44	41	43	38	34	33	28	16
Sporadic	28	35	43	37	26	35	38	38	28	24	30	21	17	20	17	11
Familial	17	23	22	32	17	17	12	18	16	17	13	17	17	13	11	5
Plexiform neurofibromas	21	23	24	28	20	25	44	41	34	33	42	33	26	25	27	16
No	11	13	13	13	10	14	21	19	20	15	17	13	8	8	6	4
Yes	10	10	11	15	10	11	23	22	14	18	25	20	18	17	21	12
ADHD diagnosis	25	27	29	27	23	29	33	36	36	32	37	34	29	29	29	17
No	24	24	20	19	10	11	19	19	18	23	24	17	13	18	20	13
Yes	1	3	9	8	13	18	14	17	18	9	13	17	16	11	9	4

Note. BASC = Behavior Assessment System for Children. NF1 = neurofibromatosis type 1. ADHD = attention-deficit/hyperactivity disorder.

eTable 13. Sample Sizes for the Associations between Each Predictor and BASC Somatization across Ages

**Predictor**	**Age Group (in years)**															
	**3.00-3.99**	**4.00-4.99**	**5.00-5.99**	**6.00-6.99**	**7.00-7.99**	**8.00-8.99**	**9.00-9.99**	**10.00-10.99**	**11.00-11.99**	**12.00-12.99**	**13.00-13.99**	**14.00-14.99**	**15.00-15.99**	**16.00-16.99**	**17.00-17.99**	**18.00-18.99**
Intercept	47	58	67	70	44	57	53	59	46	45	50	39	35	33	30	17
Sex	47	58	67	70	44	57	53	59	46	45	50	39	35	33	30	17
Male	28	34	38	37	17	30	31	31	24	32	25	20	19	19	18	10
Female	19	24	29	33	27	27	22	28	22	13	25	19	16	14	12	7
NF1 inheritance mode	45	58	65	69	43	52	50	55	44	41	43	38	34	33	28	16
Sporadic	28	35	43	37	26	35	38	38	28	24	30	21	17	20	17	11
Familial	17	23	22	32	17	17	12	17	16	17	13	17	17	13	11	5
Plexiform neurofibromas	21	23	24	28	20	25	44	41	34	33	42	33	26	25	27	16
No	11	13	13	13	10	14	21	19	20	15	17	13	8	8	6	4
Yes	10	10	11	15	10	11	23	22	14	18	25	20	18	17	21	12
ADHD diagnosis	25	27	29	27	23	29	33	36	36	32	37	34	29	29	29	17
No	24	24	20	19	10	11	19	19	18	23	24	17	13	18	20	13
Yes	1	3	9	8	13	18	14	17	18	9	13	17	16	11	9	4

Note. BASC = Behavior Assessment System for Children. NF1 = neurofibromatosis type 1. ADHD = attention-deficit/hyperactivity disorder.

eTable 14. Sample Sizes for the Associations between Each Predictor and BASC Externalizing Problems across Ages

Predictor	Age Group (in years)															
	3.00-3.99	4.00-4.99	5.00-5.99	6.00-6.99	7.00-7.99	8.00-8.99	9.00-9.99	10.00-10.99	11.00-11.99	12.00-12.99	13.00-13.99	14.00-14.99	15.00-15.99	16.00-16.99	17.00-17.99	18.00-18.99
Intercept	47	58	67	70	44	58	53	60	46	45	50	39	36	33	30	17
Sex	47	58	67	70	44	58	53	60	46	45	50	39	36	33	30	17
Male	28	34	38	37	17	31	31	31	24	32	25	21	19	19	18	10
Female	19	24	29	33	27	27	22	29	22	13	25	18	17	14	12	7
NF1 inheritance mode	45	58	65	69	43	53	50	56	44	41	43	38	35	33	28	16
Sporadic	28	35	43	37	26	36	38	38	28	24	30	22	18	20	17	11
Familial	17	23	22	32	17	17	12	18	16	17	13	16	17	13	11	5
Plexiform neurofibromas	21	23	24	28	20	26	44	41	34	33	42	33	27	25	27	16
No	11	13	13	13	10	15	21	19	20	15	17	13	9	8	6	4
Yes	10	10	11	15	10	11	23	22	14	18	25	20	18	17	21	12
ADHD diagnosis	25	27	29	27	23	30	33	36	36	32	37	34	30	29	29	17
No	24	24	20	19	10	12	19	19	18	23	24	17	14	18	20	13
Yes	1	3	9	8	13	18	14	17	18	9	13	17	16	11	9	4

Note. BASC = Behavior Assessment System for Children. NF1 = neurofibromatosis type 1. ADHD = attention-deficit/hyperactivity disorder.

eTable 15. Sample Sizes for the Associations between Each Predictor and BASC Conduct Problems across Ages

Predictor	Age Group (in years)															
	3.00-3.99	4.00-4.99	5.00-5.99	6.00-6.99	7.00-7.99	8.00-8.99	9.00-9.99	10.00-10.99	11.00-11.99	12.00-12.99	13.00-13.99	14.00-14.99	15.00-15.99	16.00-16.99	17.00-17.99	18.00-18.99
Intercept	1	2	70	44	57	53	60	46	45	50	39	35	33	30	17	1
Sex	1	2	70	44	57	53	60	46	45	50	39	35	33	30	17	1
Male	0	2	37	17	30	31	31	24	32	25	20	19	19	18	10	0
Female	1	0	33	27	27	22	29	22	13	25	19	16	14	12	7	1
NF1 inheritance mode	1	2	69	43	52	50	56	44	41	43	38	34	33	28	16	1
Sporadic	0	1	37	26	35	38	38	28	24	30	21	17	20	17	11	0
Familial	1	1	32	17	17	12	18	16	17	13	17	17	13	11	5	1
Plexiform neurofibromas	1	1	28	20	25	44	41	34	33	42	33	26	25	27	16	1
No	1	1	13	10	14	21	19	20	15	17	13	8	8	6	4	1
Yes	0	0	15	10	11	23	22	14	18	25	20	18	17	21	12	0
ADHD diagnosis	1	1	27	23	29	33	36	36	32	37	34	29	29	29	17	1
No	1	1	19	10	11	19	19	18	23	24	17	13	18	20	13	1
Yes	0	0	8	13	18	14	17	18	9	13	17	16	11	9	4	0

Note. BASC = Behavior Assessment System for Children. NF1 = neurofibromatosis type 1. ADHD = attention-deficit/hyperactivity disorder.

eTable 16. Sample Sizes for the Associations between Each Predictor and BASC Aggression across Ages

Predictor	Age Group (in years)															
	**3.00-3.99**	**4.00-4.99**	**5.00-5.99**	**6.00-6.99**	**7.00-7.99**	**8.00-8.99**	**9.00-9.99**	**10.00-10.99**	**11.00-11.99**	**12.00-12.99**	**13.00-13.99**	**14.00-14.99**	**15.00-15.99**	**16.00-16.99**	**17.00-17.99**	**18.00-18.99**
Intercept	47	58	67	70	44	57	53	60	46	45	50	38	35	33	30	17
Sex	47	58	67	70	44	57	53	60	46	45	50	38	35	33	30	17
Male	28	34	38	37	17	30	31	31	24	32	25	20	19	19	18	10
Female	19	24	29	33	27	27	22	29	22	13	25	18	16	14	12	7
NF1 inheritance mode	45	58	65	69	43	52	50	56	44	41	43	37	34	33	28	16
Sporadic	28	35	43	37	26	35	38	38	28	24	30	21	17	20	17	11
Familial	17	23	22	32	17	17	12	18	16	17	13	16	17	13	11	5
Plexiform neurofibromas	21	23	24	28	20	25	44	41	34	33	42	32	26	25	27	16
No	11	13	13	13	10	14	21	19	20	15	17	12	8	8	6	4
Yes	10	10	11	15	10	11	23	22	14	18	25	20	18	17	21	12
ADHD diagnosis	25	27	29	27	23	29	33	36	36	32	37	33	29	29	29	17
No	24	24	20	19	10	11	19	19	18	23	24	16	13	18	20	13
Yes	1	3	9	8	13	18	14	17	18	9	13	17	16	11	9	4

Note. BASC = Behavior Assessment System for Children. NF1 = neurofibromatosis type 1. ADHD = attention-deficit/hyperactivity disorder.

eTable 17. Descriptives and Correlations of CBCL Outcomes and Correlates

Variable	1	2	3	4	5	6	7	8	9	10	11	12
1. Anxious/depressed												
2. Withdrawn/depressed	.57***											
3. Somatic complaints	.45***	.43***										
4. Internalizing	.78***	.73***	.73***									
5. Rulebreaking behaviors	.39***	.36***	.21***	.41***								
6. Aggressive behaviors	.55***	.42***	.29***	.53***	.74***							
7. Externalizing	.52***	.42***	.32***	.59***	.77***	.89***						
8. Age	.08**	.13***	.05	.10**	-.04	-.07*	-.08**					
9. Sex[1]	-.02	-.11***	.00	-.06	-.06*	-.06*	-.07*	.03				
10. NF1 inheritance mode[2]	-.01	.01	-.03	-.03	.01	.05	.03	.01	.00			
11. Plexiform neurofibromas[3]	-.05	.00	-.02	-.04	.00	-.06	-.05	.07	.00	-.01		
12. ADHD diagnosis[4]	.12*	.08	.01	.12*	.17***	.19***	.26***	-.02	-.05	.00	-.08	
N	1084	1029	1083	1054	1027	1077	1057	1087	1082	758	731	427
M/*N*[5]	56.65	56.83	59.45	55.45	54.92	56.88	52.71	10.94	502	302	224	193
SD/%	8.84	8.40	9.12	11.75	6.70	8.87	11.71	3.61	0.46	0.40	0.31	0.45
% normal (T-score < 65)	80.90	82.60	74.52	76.38	88.61	81.24	83.35	-	-	-	-	-
% borderline (T-score 65-70)	9.41	8.26	9.23	9.87	6.33	8.91	7.38	-	-	-	-	-
% clinical (T-score ≥ 70)	9.69	9.14	16.25	13.76	5.06	9.84	9.27	-	-	-	-	-
% missingness	0.28	5.34	0.37	3.04	5.52	0.92	2.76	0.00	0.46	30.27	32.75	60.72

Note. CBCL = Child Behavior Checklist. ADHD = attention-deficit/hyperactivity disorder. N = valid number of observations. M = mean. SD = standard deviation. [1]female = 1; male =0. [2]familial NF1 = 1; sporadic NF1 = 0. [3]with plexiform neurofibromas = 1; no plexiform neurofibromas = 0. [4]diagnosis of ADHD = 1; no diagnosis of ADHD = 0. [5]sample size for the indicator group (e.g., females).

$^{*}p < 0.05$. $^{**}p < 0.01$. $^{***}p < 0.001$.

eTable 18. Descriptives and Correlations of BASC Outcomes and Correlates

Variable	1	2	3	4	5	6	7	8	9	10	11	12
1. Anxiety												
2. Depression	.57***											
3. Somatization	.44***	.46***										
4. Internalizing	.77***	.78***	.72***									
5. Conduct problems	.14**	.38***	.13**	.27***								
6. Aggression	.20***	.55***	.20***	.37***	.70***							
7. Externalizing	.26***	.55***	.21***	.46***	.85***	.85***						
8. Age	.23***	.13***	.12**	.15***	-.13**	-.03	-.10**					
9. Sex[1]	.08*	.06	.09*	.10**	.04	-.01	.02	-.01				
10. NF1 inheritance mode[2]	.02	.08*	-.01	.02	.18***	.10**	.14***	.01	-.01			
11. Plexiform neurofibromas[3]	-.09*	-.04	-.01	-.05	.04	-.05	-.05	.16***	-.06	.13**		
12. ADHD diagnosis[4]	.18***	.20***	.09***	.19***	.18***	.23***	.34***	.10*	-.03	.05	-.15**	
N	751	751	750	754	582	750	753	754	754	718	465	475
M/*N*[5]	53.25	54.69	55.68	55.73	50.86	50.63	53.41	9.92	339	267	257	180
SD/%	11.80	12.07	12.80	13.07	10.11	10.13	11.18	4.25	0.45	0.37	0.55	0.38
% normal (T-score < 60)	74.97	70.84	66.67	68.83	85.57	82.13	76.10	-	-	-	-	-
% at risk (T-score 60-69)	14.65	17.31	20.40	18.97	9.11	12.40	14.34	-	-	-	-	-
% clinical (T-score ≥ 70)	10.39	11.85	12.93	12.20	5.33	5.47	9.56	-	-	-	-	-
% missingness	0.40	0.40	0.37	0.53	22.81	0.53	0.13	0.00	0.00	4.77	38.33	37.00

Note. BASC = Behavior Assessment System for Children. ADHD = attention-deficit/hyperactivity disorder. N = valid number of observations. M = mean. SD = standard deviation. [1]female = 1; male =0. [2]familial NF1 = 1; sporadic NF1 = 0. [3]with plexiform neurofibromas = 1; no plexiform neurofibromas = 0. [4]diagnosis of ADHD = 1; no diagnosis of ADHD = 0. [5]sample size for the indicator group (e.g., females).

* $p < 0.05$. ** $p < 0.01$. *** $p < 0.001$.

eTable 19. Score Range by Age in the CBCL Sample

Variable	Age Group (in years)															
	**3.00-3.99**	**4.00-4.99**	**5.00-5.99**	**6.00-6.99**	**7.00-7.99**	**8.00-7.99**	**9.00-9.99**	**10.00-10.99**	**11.00-11.99**	**12.00-12.99**	**13.00-13.99**	**14.00-14.99**	**15.00-15.99**	**16.00-16.99**	**17.00-17.99**	**18.00-18.99**
Internalizing	33-71	34-85	29-80	33-79	33-80	33-89	33-80	33-84	33-87	33-86	32-81	31-77	32-81	32-78	32-76	34-76
Externalizing	42-79	35-79	28-85	33-79	32-78	30-76	30-80	30-86	30-91	32-82	33-76	32-82	33-88	32-77	32-77	34-75
Anxious/depressed	50-83	50-83	50-83	50-74	50-74	50-90	32-76	50-89	50-91	50-91	50-94	50-82	50-84	50-84	50-78	50-78
Withdrawn/depressed	50-53	50-73	50-75	50-85	50-79	50-91	50-93	50-83	50-82	50-85	50-89	50-86	50-100	50-88	50-100	50-79
Somatic complaints	50-70	50-84	50-82	50-86	50-96	50-90	47-91	50-82	50-84	50-84	50-84	50-86	50-92	50-81	50-84	50-82
Rulebreaking behaviors	50-50	50-67	50-78	50-88	50-73	50-72	50-81	50-82	50-91	50-79	50-73	50-79	50-83	50-76	50-78	50-67
Aggressive behaviors	50-79	50-91	50-97	50-89	50-85	50-82	50-89	50-95	50-95	50-88	50-86	50-86	50-97	50-78	50-79	50-84

Note. CBCL = Child Behavior Checklist.

eTable 20. Score Range by Age in the BASC Sample

Variable	Age Group (in years)															
	**3.00-3.99**	**4.00-4.99**	**5.00-5.99**	**6.00-6.99**	**7.00-7.99**	**8.00-7.99**	**9.00-9.99**	**10.00-10.99**	**11.00-11.99**	**12.00-12.99**	**13.00-13.99**	**14.00-14.99**	**15.00-15.99**	**16.00-16.99**	**17.00-17.99**	**18.00-18.99**
Internalizing	34-84	32-96	29-177	32-76	36-82	34-89	34-89	35-103	35-90	37-82	35-100	38-88	32-100	35-82	32-95	40-73
Externalizing	35-92	38-89	33-109	35-86	39-84	38-94	39-79	35-81	37-85	36-81	37-91	38-82	37-78	38-74	38-80	38-66
Anxiety	35-80	32-76	32-88	29-79	36-81	30-89	31-80	35-89	38-86	33-78	33-90	42-90	33-97	31-83	33-76	36-72
Depression	35-93	35-88	33-83	37-79	38-89	37-101	37-82	37-97	39-94	37-73	36-96	38-88	37-98	40-83	37-94	39-74
Somatization	33-79	33-78	33-88	36-70	39-83	36-98	36-92	36-102	37-91	38-80	38-102	38-108	37-104	38-83	37-103	42-76
Conduct problems	54-54	-	46-53	34-89	40-89	36-93	36-77	37-83	37-81	39-87	29-87	39-69	40-70	40-68	40-90	39-63
Aggression	37-95	38-89	35-86	35-82	39-82	37-82	37-77	37-82	40-87	37-65	38-84	39-88	37-74	40-68	37-93	38-68

Note. BASC = Behavior Assessment System for Children.

eTable 21. TVEM Estimates for CBCL Internalizing and Externalizing Problems in the Overall Sample (see Online Resource 2)

eTable 22. TVEM Estimates for CBCL Internalizing and Externalizing Problems across Sex Groups (see Online Resource 2)

eTable 23. TVEM Estimates for CBCL Internalizing and Externalizing Problems across NF1 Inheritance Mode Groups (see Online Resource 2)

eTable 24. TVEM Estimates for CBCL Internalizing and Externalizing Problems across Plexiform Neurofibromas Groups (see Online Resource 2)

eTable 25. TVEM Estimates for CBCL Internalizing and Externalizing Problems across ADHD Diagnosis Groups (see Online Resource 2)

eTable 26. TVEM Estimates for BASC Internalizing and Externalizing Problems in the Overall Sample (see Online Resource 2)

eTable 27. TVEM Estimates for BASC Internalizing and Externalizing Problems across Sex Groups (see Online Resource 2)

eTable 28. TVEM Estimates for BASC Internalizing and Externalizing Problems across NF1 Inheritance Mode Groups (see Online Resource 2)

eTable 29. TVEM Estimates for BASC Internalizing and Externalizing Problems across Plexiform Neurofibromas Groups (see Online Resource 2)

eTable 30. TVEM Estimates for BASC Internalizing and Externalizing Problems across ADHD Diagnosis Groups (see Online Resource 2)

eTable 31. Model Fit Comparison Across Random Effect Specifications in the CBCL Sample

**Outcome**	**Model**	**Random Effects Specification**	**AIC**	**BIC**	**Δ AIC**	**Δ BIC**	**Likelihood Ratio**	***p* value**
Internalizing	Intercept	Participant only	7986.194	8010.996	-	-	-	-
Internalizing	Intercept	Participant + Site	7985.369	8015.132	-0.825	4.136	1.516	0.218
Internalizing	Male	Participant only	4236.247	4257.887	-	-	-	-
Internalizing	Male	Participant + Site	4238.247	4264.215	2.000	6.328	0.000	1.000
Internalizing	Female	Participant only	3713.603	3734.564	-	-	-	-
Internalizing	Female	Participant + Site	3709.519	3734.673	-4.084	0.108	4.647	0.031
Internalizing	Sporadic NF1	Participant only	3395.038	3415.618	-	-	-	-
Internalizing	Sporadic NF1	Participant + Site	3392.690	3417.386	-2.348	1.768	2.706	0.100
Internalizing	Familial NF1	Participant only	2290.082	2308.584	-	-	-	-
Internalizing	Familial NF1	Participant + Site	2292.053	2314.255	1.971	5.671	0.000	1.000
Internalizing	No PNs	Participant only	3811.018	3832.131	-	-	-	-
Internalizing	No PNs	Participant + Site	3812.926	3838.261	1.907	6.130	0.000	1.000
Internalizing	PNs	Participant only	1690.058	1707.072	-	-	-	-
Internalizing	PNs	Participant + Site	1686.679	1707.095	-3.379	0.024	2.286	0.131
Internalizing	No ADHD	Participant only	1714.891	1731.904	-	-	-	-
Internalizing	No ADHD	Participant + Site	1711.800	1732.216	-3.090	0.312	3.924	0.048
Internalizing	ADHD	Participant only	1360.343	1376.252	-	-	-	-
Internalizing	ADHD	Participant + Site	1362.343	1381.433	2.000	5.182	0.000	1.000
Externalizing	Intercept	Participant only	7907.243	7932.059	-	-	-	-
Externalizing	Intercept	Participant + Site	7908.880	7938.659	1.637	6.600	0.000	1.000
Externalizing	Male	Participant only	4220.921	4242.579	-	-	-	-
Externalizing	Male	Participant + Site	4222.921	4248.910	2.000	6.332	0.000	1.000
Externalizing	Female	Participant only	3643.497	3664.469	-	-	-	-

**Outcome**	**Model**	**Random Effects Specification**	**AIC**	**BIC**	**Δ AIC**	**Δ BIC**	**Likelihood Ratio**	***p* value**
Externalizing	Female	Participant + Site	3644.237	3669.403	0.740	4.934	0.206	0.650
Externalizing	Sporadic NF1	Participant only	3321.517	3342.107	-	-	-	-
Externalizing	Sporadic NF1	Participant + Site	3321.766	3346.475	0.249	4.367	0.570	0.450
Externalizing	Familial NF1	Participant only	2307.034	2325.569	-	-	-	-
Externalizing	Familial NF1	Participant + Site	2309.034	2331.277	2.000	5.707	0.000	1.000
Externalizing	No PNs	Participant only	3794.423	3815.555	-	-	-	-
Externalizing	No PNs	Participant + Site	3796.423	3821.782	2.000	6.227	0.000	1.000
Externalizing	PNs	Participant only	1673.435	1690.471	-	-	-	-
Externalizing	PNs	Participant + Site	1675.435	1695.878	2.000	5.407	0.000	1.000
Externalizing	No ADHD	Participant only	1683.068	1700.082	-	-	-	-
Externalizing	No ADHD	Participant + Site	1680.015	1700.431	-3.053	0.349	3.957	0.047
Externalizing	ADHD	Participant only	1330.127	1346.035	-	-	-	-
Externalizing	ADHD	Participant + Site	1329.506	1348.596	-0.621	2.561	0.992	0.319
Anxious/depressed	Intercept	Participant only	7474.845	7499.787	-	-	-	-
Anxious/depressed	Intercept	Participant + Site	7472.313	7502.244	-2.532	2.456	3.264	0.071
Anxious/depressed	Male	Participant only	3994.494	4016.300	-	-	-	-
Anxious/depressed	Male	Participant + Site	3996.494	4022.662	2.000	6.361	0.000	1.000
Anxious/depressed	Female	Participant only	3451.534	3472.607	-	-	-	-
Anxious/depressed	Female	Participant + Site	3444.909	3470.197	-6.624	-2.410	7.411	0.006
Anxious/depressed	Sporadic NF1	Participant only	3093.494	3114.096	-	-	-	-
Anxious/depressed	Sporadic NF1	Participant + Site	3090.712	3115.434	-2.782	1.338	3.124	0.077
Anxious/depressed	Familial NF1	Participant only	2081.537	2100.073	-	-	-	-
Anxious/depressed	Familial NF1	Participant + Site	2083.537	2105.780	2.000	5.707	0.000	1.000
Anxious/depressed	No PNs	Participant only	3470.873	3492.006	-	-	-	-

**Outcome**	**Model**	**Random Effects Specification**	**AIC**	**BIC**	**Δ AIC**	**Δ BIC**	**Likelihood Ratio**	***p* value**
Anxious/depressed	No PNs	Participant + Site	3472.603	3497.962	1.730	5.957	0.000	1.000
Anxious/depressed	PNs	Participant only	1508.580	1525.615	-	-	-	-
Anxious/depressed	PNs	Participant + Site	1505.816	1526.259	-2.764	0.643	2.331	0.127
Anxious/depressed	No ADHD	Participant only	1601.556	1618.811	-	-	-	-
Anxious/depressed	No ADHD	Participant + Site	1601.080	1621.787	-0.476	2.975	1.580	0.209
Anxious/depressed	ADHD	Participant only	1394.836	1411.149	-	-	-	-
Anxious/depressed	ADHD	Participant + Site	1396.836	1416.412	2.000	5.263	0.000	1.000
Withdrawn/depressed	Intercept	Participant only	7130.107	7154.789	-	-	-	-
Withdrawn/depressed	Intercept	Participant + Site	7130.075	7159.693	-0.033	4.904	0.860	0.354
Withdrawn/depressed	Male	Participant only	3773.048	3794.478	-	-	-	-
Withdrawn/depressed	Male	Participant + Site	3775.048	3800.764	2.000	6.286	0.000	1.000
Withdrawn/depressed	Female	Participant only	3320.488	3341.440	-	-	-	-
Withdrawn/depressed	Female	Participant + Site	3317.909	3343.051	-2.579	1.611	3.248	0.072
Withdrawn/depressed	Sporadic NF1	Participant only	3013.947	3034.347	-	-	-	-
Withdrawn/depressed	Sporadic NF1	Participant + Site	3015.597	3040.077	1.650	5.730	0.000	1.000
Withdrawn/depressed	Familial NF1	Participant only	2040.153	2058.502	-	-	-	-
Withdrawn/depressed	Familial NF1	Participant + Site	2040.648	2062.667	0.494	4.164	0.356	0.551
Withdrawn/depressed	No PNs	Participant only	3392.470	3413.380	-	-	-	-
Withdrawn/depressed	No PNs	Participant + Site	3393.601	3418.694	1.131	5.313	0.000	1.000
Withdrawn/depressed	PNs	Participant only	1486.190	1503.020	-	-	-	-
Withdrawn/depressed	PNs	Participant + Site	1487.707	1507.903	1.517	4.883	0.000	1.000
Withdrawn/depressed	No ADHD	Participant only	1484.019	1500.941	-	-	-	-
Withdrawn/depressed	No ADHD	Participant + Site	1479.446	1499.753	-4.573	-1.188	5.304	0.021
Withdrawn/depressed	ADHD	Participant only	1260.192	1276.073	-	-	-	-

**Outcome**	**Model**	**Random Effects Specification**	**AIC**	**BIC**	**Δ AIC**	**Δ BIC**	**Likelihood Ratio**	***p* value**
Withdrawn/depressed	ADHD	Participant + Site	1262.192	1281.249	2.000	5.176	0.000	1.000
Somatic complaints	Intercept	Participant only	7728.689	7753.627	-	-	-	-
Somatic complaints	Intercept	Participant + Site	7727.586	7757.511	-1.104	3.884	1.615	0.204
Somatic complaints	Male	Participant only	4119.505	4141.303	-	-	-	-
Somatic complaints	Male	Participant + Site	4121.505	4147.662	2.000	6.359	0.000	1.000
Somatic complaints	Female	Participant only	3572.911	3593.984	-	-	-	-
Somatic complaints	Female	Participant + Site	3573.226	3598.514	0.315	4.530	0.485	0.486
Somatic complaints	Sporadic NF1	Participant only	3246.984	3267.575	-	-	-	-
Somatic complaints	Sporadic NF1	Participant + Site	3247.322	3272.031	0.338	4.456	0.317	0.573
Somatic complaints	Familial NF1	Participant only	2144.427	2162.963	-	-	-	-
Somatic complaints	Familial NF1	Participant + Site	2146.427	2168.670	2.000	5.707	0.000	1.000
Somatic complaints	No PNs	Participant only	3602.317	3623.440	-	-	-	-
Somatic complaints	No PNs	Participant + Site	3604.317	3629.664	2.000	6.225	0.000	1.000
Somatic complaints	PNs	Participant only	1608.197	1625.233	-	-	-	-
Somatic complaints	PNs	Participant + Site	1608.854	1629.297	0.657	4.064	0.000	1.000
Somatic complaints	No ADHD	Participant only	1654.806	1672.061	-	-	-	-
Somatic complaints	No ADHD	Participant + Site	1653.100	1673.806	-1.706	1.745	2.729	0.099
Somatic complaints	ADHD	Participant only	1379.060	1395.347	-	-	-	-
Somatic complaints	ADHD	Participant + Site	1380.746	1400.291	1.687	4.944	0.000	1.000
Rulebreaking behaviors	Intercept	Participant only	6658.248	6682.920	-	-	-	-
Rulebreaking behaviors	Intercept	Participant + Site	6660.143	6689.750	1.895	6.829	0.000	1.000

**Outcome**	**Model**	**Random Effects Specification**	**AIC**	**BIC**	**Δ AIC**	**Δ BIC**	**Likelihood Ratio**	***p* value**
Rulebreaking behaviors	Male	Participant only	3535.989	3557.419	-	-	-	-
Rulebreaking behaviors	Male	Participant + Site	3537.989	3563.705	2.000	6.286	0.000	1.000
Rulebreaking behaviors	Female	Participant only	3083.942	3104.874	-	-	-	-
Rulebreaking behaviors	Female	Participant + Site	3085.784	3110.901	1.842	6.028	0.000	1.000
Rulebreaking behaviors	Sporadic NF1	Participant only	2807.604	2828.003	-	-	-	-
Rulebreaking behaviors	Sporadic NF1	Participant + Site	2808.302	2832.782	0.698	4.778	0.160	0.689
Rulebreaking behaviors	Familial NF1	Participant only	1906.569	1924.884	-	-	-	-
Rulebreaking behaviors	Familial NF1	Participant + Site	1908.569	1930.547	2.000	5.663	0.000	1.000
Rulebreaking behaviors	No PNs	Participant only	3132.879	3153.779	-	-	-	-
Rulebreaking behaviors	No PNs	Participant + Site	3134.879	3159.959	2.000	6.180	0.000	1.000
Rulebreaking behaviors	PNs	Participant only	1429.726	1446.556	-	-	-	-
Rulebreaking behaviors	PNs	Participant + Site	1431.422	1451.618	1.696	5.062	0.000	1.000

Outcome	Model	Random Effects Specification	AIC	BIC	Δ AIC	Δ BIC	Likelihood Ratio	*p* value
Rulebreaking behaviors	No ADHD	Participant only	1382.515	1399.415	-	-	-	-
Rulebreaking behaviors	No ADHD	Participant + Site	1384.123	1404.402	1.607	4.987	0.060	0.806
Rulebreaking behaviors	ADHD	Participant only	1178.632	1194.513	-	-	-	-
Rulebreaking behaviors	ADHD	Participant + Site	1173.025	1192.082	-5.606	-2.430	5.248	0.022
Aggressive behaviors	Intercept	Participant only	7471.318	7496.228	-	-	-	-
Aggressive behaviors	Intercept	Participant + Site	7471.546	7501.437	0.227	5.209	0.496	0.481
Aggressive behaviors	Male	Participant only	4065.142	4086.905	-	-	-	-
Aggressive behaviors	Male	Participant + Site	4067.142	4093.258	2.000	6.353	0.000	1.000
Aggressive behaviors	Female	Participant only	3350.897	3371.950	-	-	-	-
Aggressive behaviors	Female	Participant + Site	3350.178	3375.441	-0.719	3.491	1.405	0.236
Aggressive behaviors	Sporadic NF1	Participant only	3059.397	3079.933	-	-	-	-
Aggressive behaviors	Sporadic NF1	Participant + Site	3059.144	3083.786	-0.253	3.854	0.910	0.340
Aggressive behaviors	Familial NF1	Participant only	2145.541	2164.060	-	-	-	-
Aggressive behaviors	Familial NF1	Participant + Site	2147.541	2169.764	2.000	5.704	0.000	1.000
Aggressive behaviors	No PNs	Participant only	3489.851	3510.914	-	-	-	-
Aggressive behaviors	No PNs	Participant + Site	3491.851	3517.127	2.000	6.213	0.000	1.000
Aggressive behaviors	PNs	Participant only	1550.051	1567.086	-	-	-	-
Aggressive behaviors	PNs	Participant + Site	1551.861	1572.304	1.810	5.218	0.000	1.000
Aggressive behaviors	No ADHD	Participant only	1535.796	1552.942	-	-	-	-
Aggressive behaviors	No ADHD	Participant + Site	1530.832	1551.408	-4.963	-1.534	5.611	0.018

**Outcome**	**Model**	**Random Effects Specification**	**AIC**	**BIC**	**Δ AIC**	**Δ BIC**	**Likelihood Ratio**	***p* value**
Aggressive behaviors	ADHD	Participant only	1372.370	1388.632	-	-	-	-
Aggressive behaviors	ADHD	Participant + Site	1371.594	1391.108	-0.776	2.476	0.956	0.328

Note. CBCL = Child Behavior Checklist. AIC = Akaike information criterion, BIC = Bayesian information criterion. NF1 = neurofibromatosis type 1. PNs = plexiform neurofibromas. ADHD = attention-deficit/hyperactivity disorder. Significant p value indicates significant improvement in model fit. Models with random effects specified for participants only are main analyses accounting for participant-level clustering. Models with random effects specified for both participants and sites are sensitivity analyses accounting for participant- and site-level clustering.

eTable 32. Model Fit Comparison Across Random Effect Specifications in the BASC Sample

**Outcome**	**Model**	**Random Effects Specification**	**AIC**	**BIC**	**Δ AIC**	**Δ BIC**	**Likelihood Ratio**	***p* value**
Internalizing	Intercept	Participant only	5846.027	5869.154	-	-	-	-
Internalizing	Intercept	Participant + Site	5847.131	5874.883	1.104	5.730	0.363	0.547
Internalizing	Male	Participant only	3142.191	3162.332	-	-	-	-
Internalizing	Male	Participant + Site	3144.191	3168.361	2.000	6.028	0.000	1.000
Internalizing	Female	Participant only	2690.807	2709.937	-	-	-	-
Internalizing	Female	Participant + Site	2692.747	2715.703	1.939	5.765	0.000	1.000
Internalizing	Sporadic NF1	Participant only	3507.687	3528.244	-	-	-	-
Internalizing	Sporadic NF1	Participant + Site	3509.574	3534.243	1.887	5.998	0.000	1.000
Internalizing	Familial NF1	Participant only	2082.062	2099.998	-	-	-	-
Internalizing	Familial NF1	Participant + Site	2084.062	2105.585	2.000	5.587	0.000	1.000
Internalizing	No PNs	Participant only	1718.426	1735.114	-	-	-	-
Internalizing	No PNs	Participant + Site	1720.296	1740.321	1.870	5.207	0.000	1.000
Internalizing	PNs	Participant only	1935.304	1953.049	-	-	-	-
Internalizing	PNs	Participant + Site	1936.930	1958.225	1.627	5.176	0.000	1.000
Internalizing	No ADHD	Participant only	2215.852	2234.287	-	-	-	-
Internalizing	No ADHD	Participant + Site	2217.852	2239.974	2.000	5.687	0.000	1.000
Internalizing	ADHD	Participant only	1470.166	1486.131	-	-	-	-
Internalizing	ADHD	Participant + Site	1472.166	1491.324	2.000	5.193	0.000	1.000
Externalizing	Intercept	Participant only	5549.791	5572.911	-	-	-	-
Externalizing	Intercept	Participant + Site	5543.089	5570.834	-6.701	-2.077	7.145	0.008
Externalizing	Male	Participant only	3064.324	3084.466	-	-	-	-
Externalizing	Male	Participant + Site	3064.515	3088.685	0.191	4.219	1.005	0.316
Externalizing	Female	Participant only	2484.575	2503.690	-	-	-	-
Externalizing	Female	Participant + Site	2480.349	2503.287	-4.226	-0.402	4.770	0.029

Outcome	Model	Random Effects Specification	AIC	BIC	Δ AIC	Δ BIC	Likelihood Ratio	*p* value
Externalizing	Sporadic NF1	Participant only	3290.857	3311.414	-	-	-	-
Externalizing	Sporadic NF1	Participant + Site	3291.008	3315.677	0.151	4.263	1.083	0.298
Externalizing	Familial NF1	Participant only	2005.613	2023.530	-	-	-	-
Externalizing	Familial NF1	Participant + Site	2004.986	2026.487	-0.627	2.957	1.724	0.189
Externalizing	No PNs	Participant only	1588.139	1604.802	-	-	-	-
Externalizing	No PNs	Participant + Site	1590.139	1610.135	2.000	5.333	0.000	1.000
Externalizing	PNs	Participant only	1820.161	1837.906	-	-	-	-
Externalizing	PNs	Participant + Site	1819.806	1841.101	-0.354	3.195	1.069	0.301
Externalizing	No ADHD	Participant only	2050.139	2068.557	-	-	-	-
Externalizing	No ADHD	Participant + Site	2052.139	2074.240	2.000	5.684	0.000	1.000
Externalizing	ADHD	Participant only	1342.027	1357.991	-	-	-	-
Externalizing	ADHD	Participant + Site	1344.027	1363.184	2.000	5.193	0.000	1.000
Anxiety	Intercept	Participant only	5663.920	5687.027	-	-	-	-
Anxiety	Intercept	Participant + Site	5665.469	5693.197	1.549	6.170	0.087	0.768
Anxiety	Male	Participant only	3090.049	3110.166	-	-	-	-
Anxiety	Male	Participant + Site	3092.049	3116.190	2.000	6.023	0.000	1.000
Anxiety	Female	Participant only	2576.206	2595.321	-	-	-	-
Anxiety	Female	Participant + Site	2577.780	2600.718	1.574	5.397	0.032	0.857
Anxiety	Sporadic NF1	Participant only	3357.131	3377.655	-	-	-	-
Anxiety	Sporadic NF1	Participant + Site	3358.323	3382.952	1.193	5.297	0.229	0.632
Anxiety	Familial NF1	Participant only	2051.302	2069.238	-	-	-	-
Anxiety	Familial NF1	Participant + Site	2053.302	2074.826	2.000	5.587	0.000	1.000
Anxiety	No PNs	Participant only	1606.357	1622.972	-	-	-	-
Anxiety	No PNs	Participant + Site	1608.312	1628.250	1.955	5.278	0.000	1.000
Anxiety	PNs	Participant only	1921.087	1938.833	-	-	-	-

Outcome	Model	Random Effects Specification	AIC	BIC	Δ AIC	Δ BIC	Likelihood Ratio	*p* value
Anxiety	PNs	Participant + Site	1923.087	1944.382	2.000	5.549	0.000	1.000
Anxiety	No ADHD	Participant only	2177.556	2195.940	-	-	-	-
Anxiety	No ADHD	Participant + Site	2179.556	2201.617	2.000	5.677	0.000	1.000
Anxiety	ADHD	Participant only	1379.884	1395.849	-	-	-	-
Anxiety	ADHD	Participant + Site	1381.884	1401.042	2.000	5.193	0.000	1.000
Depression	Intercept	Participant only	5720.752	5743.859	-	-	-	-
Depression	Intercept	Participant + Site	5720.885	5748.613	0.133	4.754	1.070	0.301
Depression	Male	Participant only	3114.912	3135.030	-	-	-	-
Depression	Male	Participant + Site	3116.876	3141.017	1.964	5.987	0.000	1.000
Depression	Female	Participant only	2607.446	2626.561	-	-	-	-
Depression	Female	Participant + Site	2609.238	2632.176	1.793	5.616	0.000	1.000
Depression	Sporadic NF1	Participant only	3397.161	3417.685	-	-	-	-
Depression	Sporadic NF1	Participant + Site	3398.318	3422.946	1.156	5.261	0.307	0.579
Depression	Familial NF1	Participant only	2065.771	2083.707	-	-	-	-
Depression	Familial NF1	Participant + Site	2067.771	2089.294	2.000	5.587	0.000	1.000
Depression	No PNs	Participant only	1601.106	1617.721	-	-	-	-
Depression	No PNs	Participant + Site	1601.380	1621.318	0.274	3.597	0.662	0.416
Depression	PNs	Participant only	1915.042	1932.787	-	-	-	-
Depression	PNs	Participant + Site	1917.042	1938.336	2.000	5.549	0.000	1.000
Depression	No ADHD	Participant only	2167.542	2185.926	-	-	-	-
Depression	No ADHD	Participant + Site	2169.542	2191.603	2.000	5.677	0.000	1.000
Depression	ADHD	Participant only	1389.232	1405.196	-	-	-	-
Depression	ADHD	Participant + Site	1391.232	1410.389	2.000	5.193	0.000	1.000
Somatization	Intercept	Participant only	5792.546	5815.647	-	-	-	-
Somatization	Intercept	Participant + Site	5794.035	5821.756	1.489	6.109	0.104	0.747

Outcome	Model	Random Effects Specification	AIC	BIC	Δ AIC	Δ BIC	Likelihood Ratio	*p* value
Somatization	Male	Participant only	3200.792	3220.909	-	-	-	-
Somatization	Male	Participant + Site	3202.678	3226.818	1.886	5.910	0.000	1.000
Somatization	Female	Participant only	2585.358	2604.458	-	-	-	-
Somatization	Female	Participant + Site	2587.358	2610.278	2.000	5.820	0.000	1.000
Somatization	Sporadic NF1	Participant only	3420.651	3441.175	-	-	-	-
Somatization	Sporadic NF1	Participant + Site	3422.651	3447.280	2.000	6.105	0.000	1.000
Somatization	Familial NF1	Participant only	2093.683	2111.601	-	-	-	-
Somatization	Familial NF1	Participant + Site	2093.014	2114.515	-0.669	2.914	1.596	0.206
Somatization	No PNs	Participant only	1649.086	1665.701	-	-	-	-
Somatization	No PNs	Participant + Site	1651.086	1671.024	2.000	5.323	0.000	1.000
Somatization	PNs	Participant only	1959.257	1977.003	-	-	-	-
Somatization	PNs	Participant + Site	1961.047	1982.341	1.789	5.338	0.000	1.000
Somatization	No ADHD	Participant only	2209.973	2228.357	-	-	-	-
Somatization	No ADHD	Participant + Site	2211.973	2234.034	2.000	5.677	0.000	1.000
Somatization	ADHD	Participant only	1447.447	1463.412	-	-	-	-
Somatization	ADHD	Participant + Site	1449.447	1468.605	2.000	5.193	0.000	1.000
Conduct problems	Intercept	Participant only	4228.360	4250.192	-	-	-	-
Conduct problems	Intercept	Participant + Site	4230.360	4256.559	2.000	6.367	0.000	1.000
Conduct problems	Male	Participant only	2254.891	2273.654	-	-	-	-
Conduct problems	Male	Participant + Site	2256.891	2279.406	2.000	5.753	0.000	1.000
Conduct problems	Female	Participant only	1971.584	1989.520	-	-	-	-
Conduct problems	Female	Participant + Site	1971.719	1993.243	0.135	3.722	0.848	0.357
Conduct problems	Sporadic NF1	Participant only	2432.669	2451.857	-	-	-	-
Conduct problems	Sporadic NF1	Participant + Site	2434.573	2457.599	1.904	5.742	0.000	1.000
Conduct problems	Familial NF1	Participant only	1556.218	1572.882	-	-	-	-

Outcome	Model	Random Effects Specification	AIC	BIC	Δ AIC	Δ BIC	Likelihood Ratio	*p* value
Conduct problems	Familial NF1	Participant + Site	1558.208	1578.205	1.990	5.323	0.000	1.000
Conduct problems	No PNs	Participant only	1252.256	1267.935	-	-	-	-
Conduct problems	No PNs	Participant + Site	1254.256	1273.071	2.000	5.136	0.000	1.000
Conduct problems	PNs	Participant only	1589.650	1606.753	-	-	-	-
Conduct problems	PNs	Participant + Site	1590.903	1611.427	1.253	4.674	0.024	0.878
Conduct problems	No ADHD	Participant only	1576.425	1593.528	-	-	-	-
Conduct problems	No ADHD	Participant + Site	1578.425	1598.949	2.000	5.421	0.000	1.000
Conduct problems	ADHD	Participant only	1206.083	1221.673	-	-	-	-
Conduct problems	ADHD	Participant + Site	1208.083	1226.791	2.000	5.118	0.000	1.000
Aggression	Intercept	Participant only	5463.449	5486.549	-	-	-	-
Aggression	Intercept	Participant + Site	5460.885	5488.606	-2.564	2.056	3.403	0.065
Aggression	Male	Participant only	3059.426	3079.543	-	-	-	-
Aggression	Male	Participant + Site	3059.753	3083.894	0.327	4.350	0.932	0.334
Aggression	Female	Participant only	2399.041	2418.141	-	-	-	-
Aggression	Female	Participant + Site	2400.253	2423.173	1.212	5.032	0.229	0.632
Aggression	Sporadic NF1	Participant only	3232.986	3253.510	-	-	-	-
Aggression	Sporadic NF1	Participant + Site	3234.678	3259.307	1.692	5.796	0.029	0.864
Aggression	Familial NF1	Participant only	1975.188	1993.105	-	-	-	-
Aggression	Familial NF1	Participant + Site	1975.531	1997.032	0.343	3.927	0.959	0.327
Aggression	No PNs	Participant only	1530.413	1547.003	-	-	-	-
Aggression	No PNs	Participant + Site	1532.413	1552.322	2.000	5.318	0.000	1.000
Aggression	PNs	Participant only	1791.898	1809.643	-	-	-	-
Aggression	PNs	Participant + Site	1793.767	1815.062	1.870	5.419	0.000	1.000
Aggression	No ADHD	Participant only	2010.087	2028.453	-	-	-	-
Aggression	No ADHD	Participant + Site	2012.087	2034.126	2.000	5.673	0.000	1.000

**Outcome**	**Model**	**Random Effects Specification**	**AIC**	**BIC**	**Δ AIC**	**Δ BIC**	**Likelihood Ratio**	***p* value**
Aggression	ADHD	Participant only	1341.854	1357.819	-	-	-	-
Aggression	ADHD	Participant + Site	1343.854	1363.012	2.000	5.193	0.000	1.000

Note. BASC = Behavior Assessment System for Children. AIC = Akaike information criterion, BIC = Bayesian information criterion. NF1 = neurofibromatosis type 1. PNs = plexiform neurofibromas. ADHD = attention-deficit/hyperactivity disorder. Significant p value indicates significant improvement in model fit. Models with random effects specified for participants only are main analyses accounting for participant-level clustering. Models with random effects specified for both participants and sites are sensitivity analyses accounting for participant- and site-level clustering.

eFigure 1. Age Trends of Subdomains of Internalizing Problems

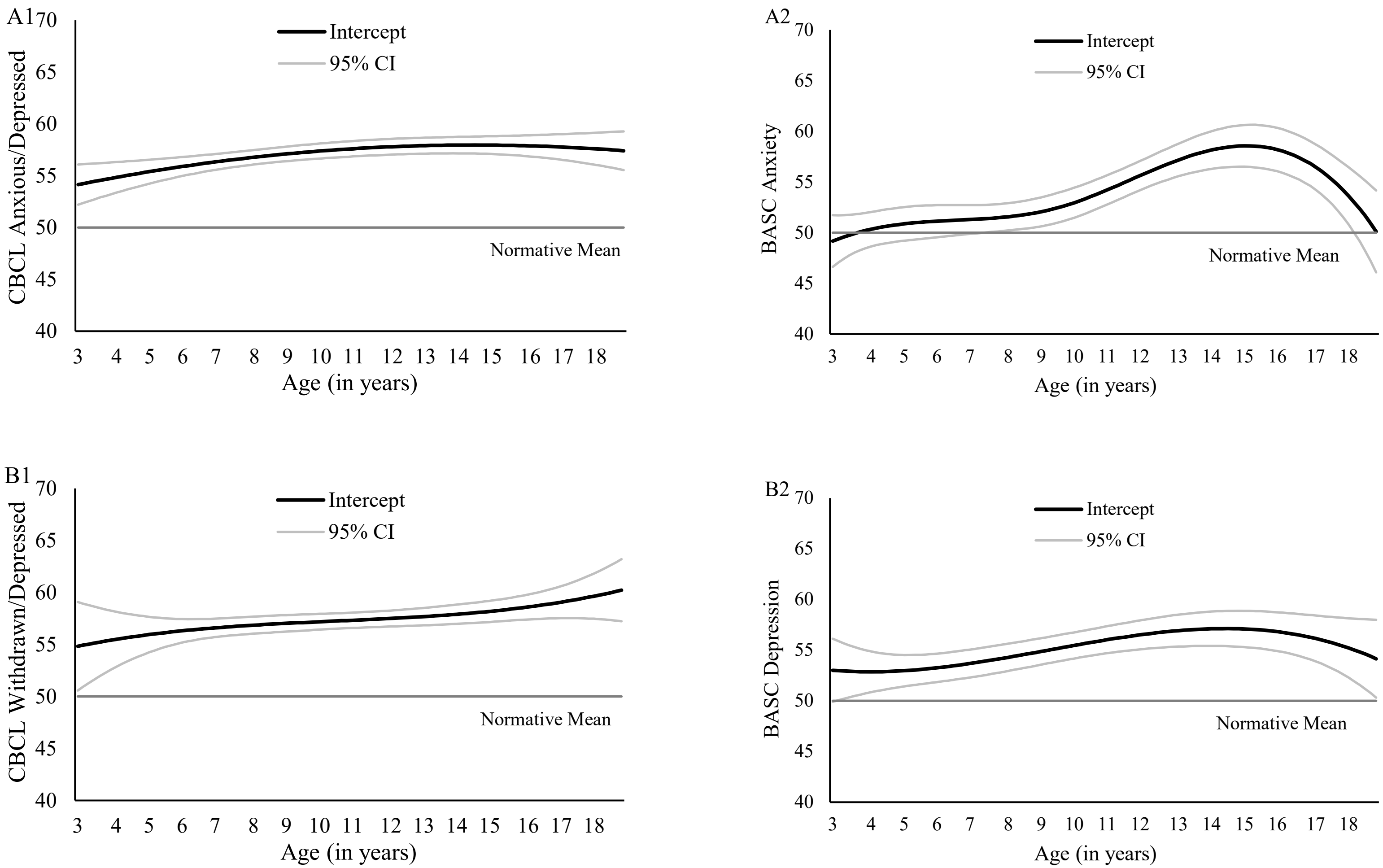

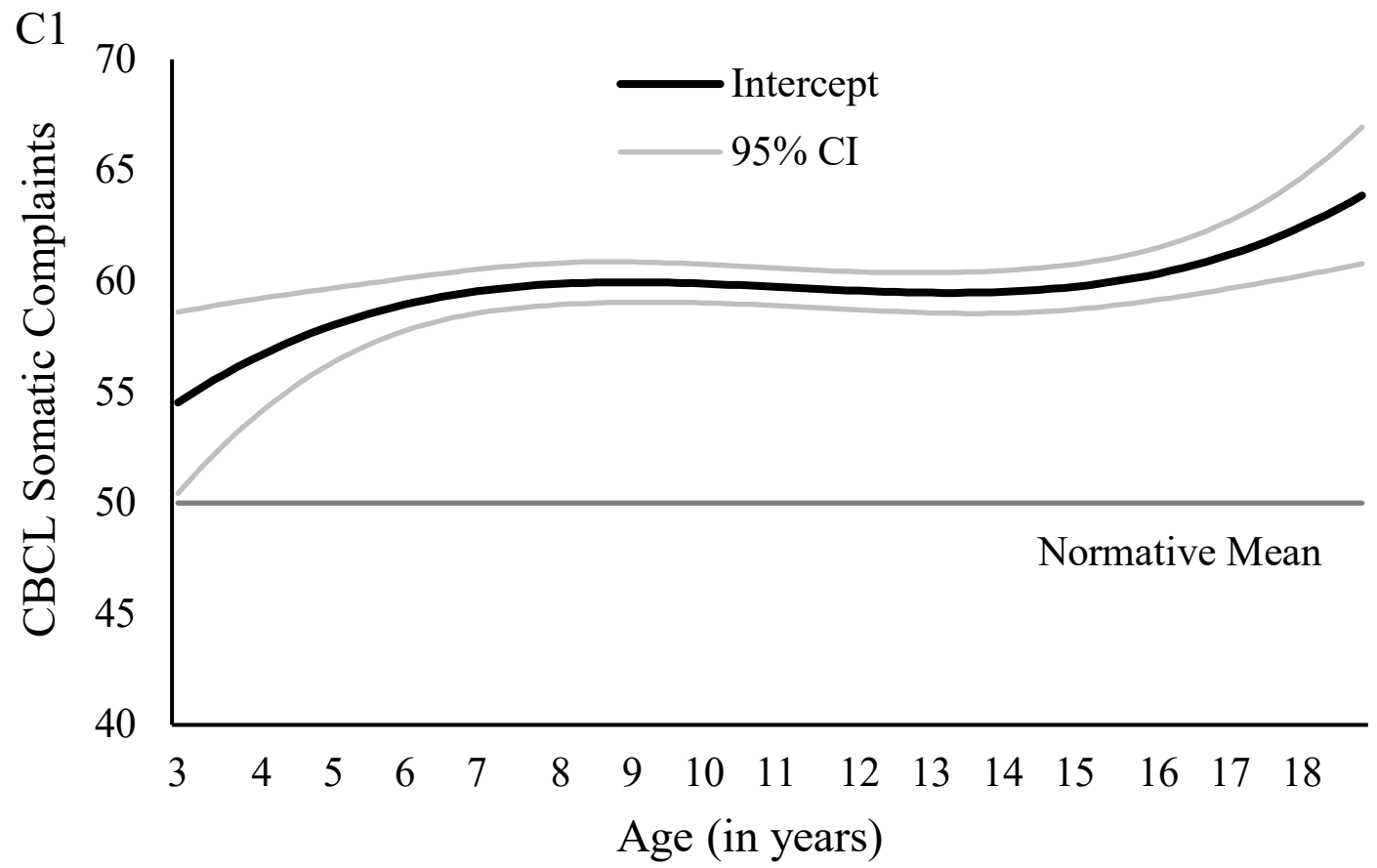

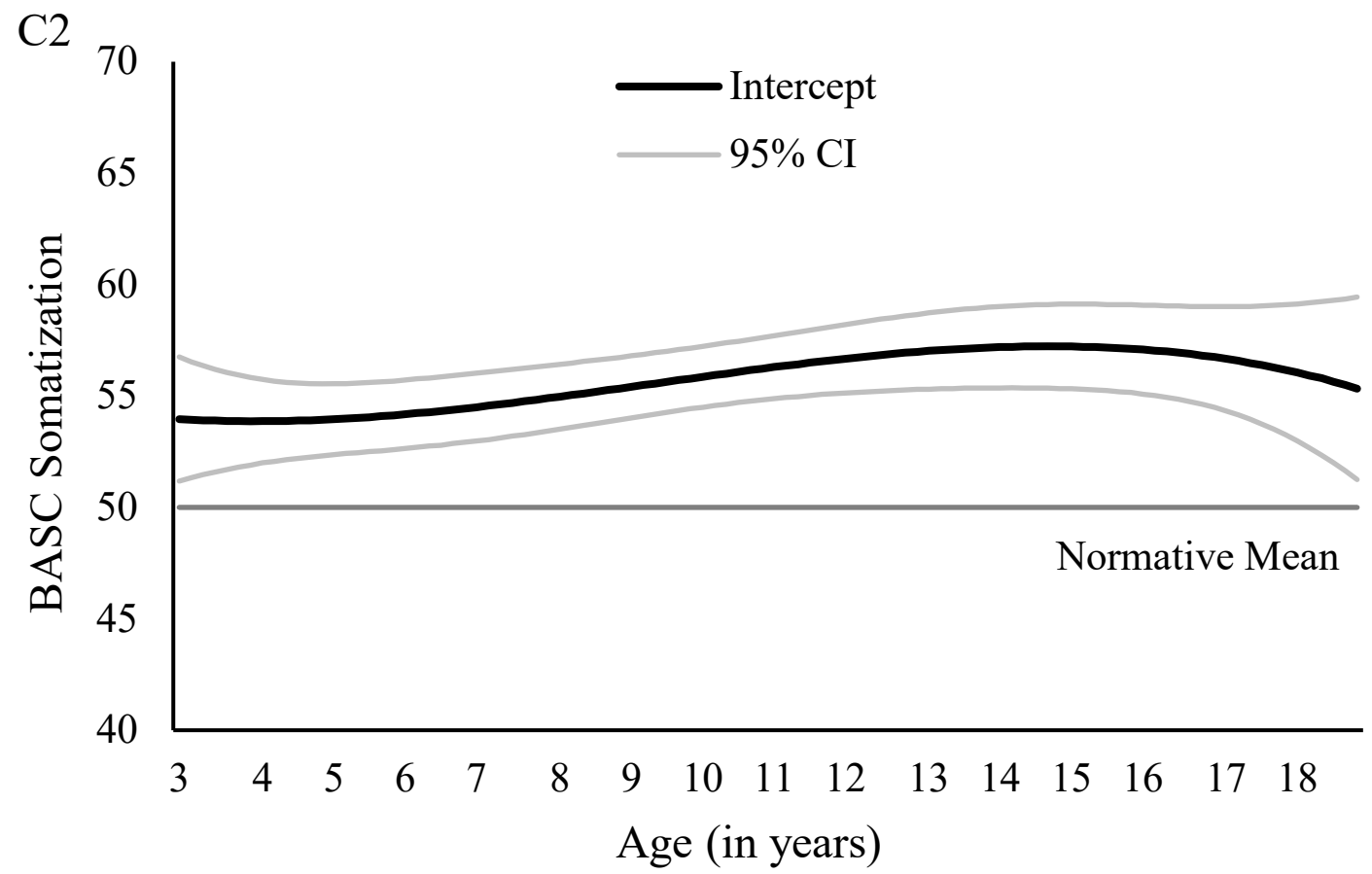

Note. Outcomes include Child Behavior Checklist (CBCL) anxious/depressed (A1), CBCL withdrawn/depressed (B1), CBCL somatic complaints (C2), Behavior Assessment System for Children (BASC) anxiety (A2), BASC depression (B2), and BASC somatization (C2). Internalizing subdomains are presented using standardized T scores (M_{norm} = 50, SD_{norm} = 10). Significant deviations from the normative sample are indicated by 95% confidence intervals (CIs) that do not include the normative mean of 50.

eFigure 2. Age Trends of Subdomains of Externalizing Problems

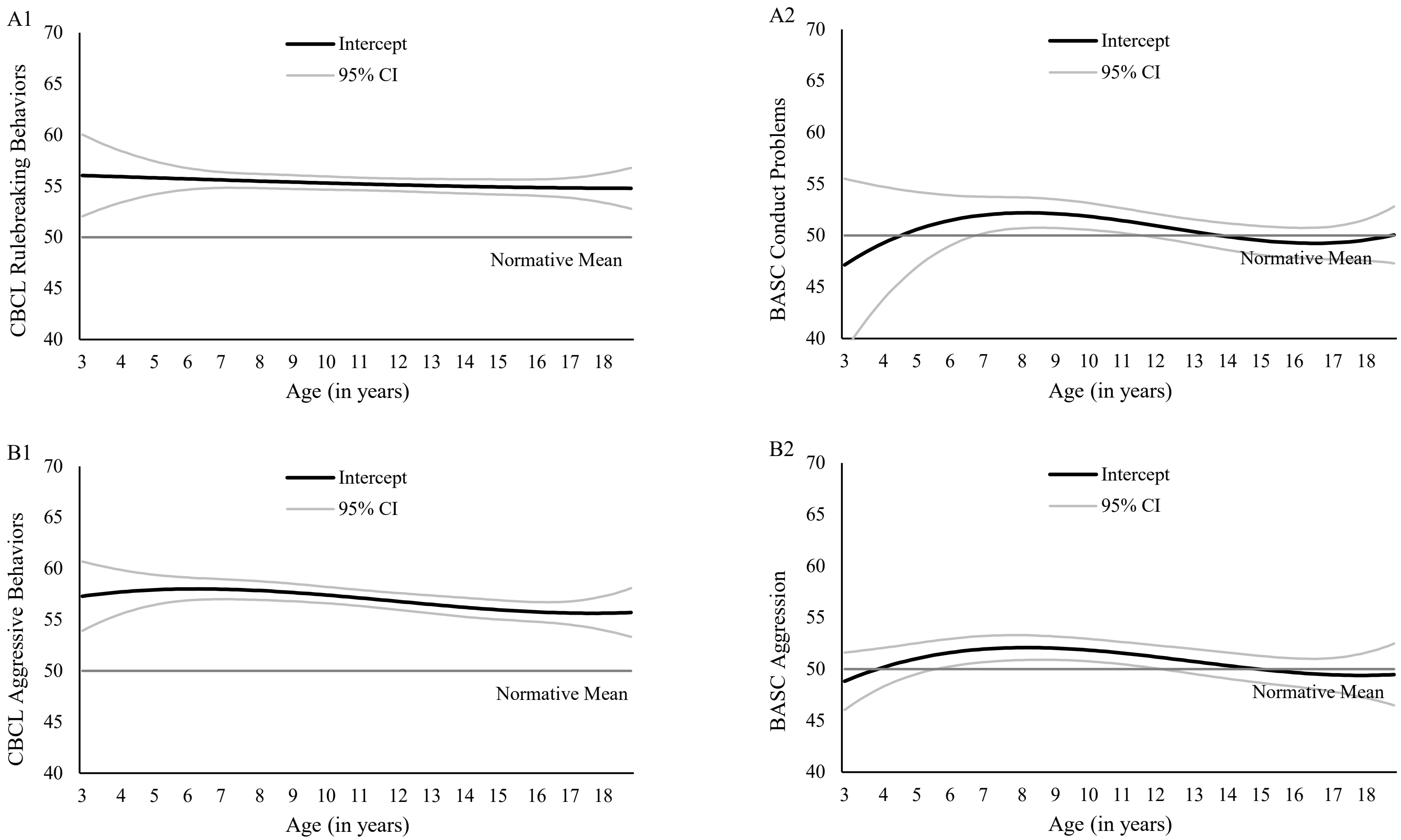

Note. Outcomes include Child Behavior Checklist (CBCL) rulebreaking behaviors (A1), CBCL aggressive behaviors (B1), Behavior Assessment System for Children (BASC) conduct problems (A2), and BASC aggression (B2). Externalizing subdomains are presented using standardized T scores ($M_{norm} = 50$, $SD_{norm} = 10$). Significant deviations from the normative sample are indicated by 95% confidence intervals (CIs) that do not include the normative mean of 50.

eFigure 3. Age Trends of Subdomains of Internalizing Problems across Sex Groups

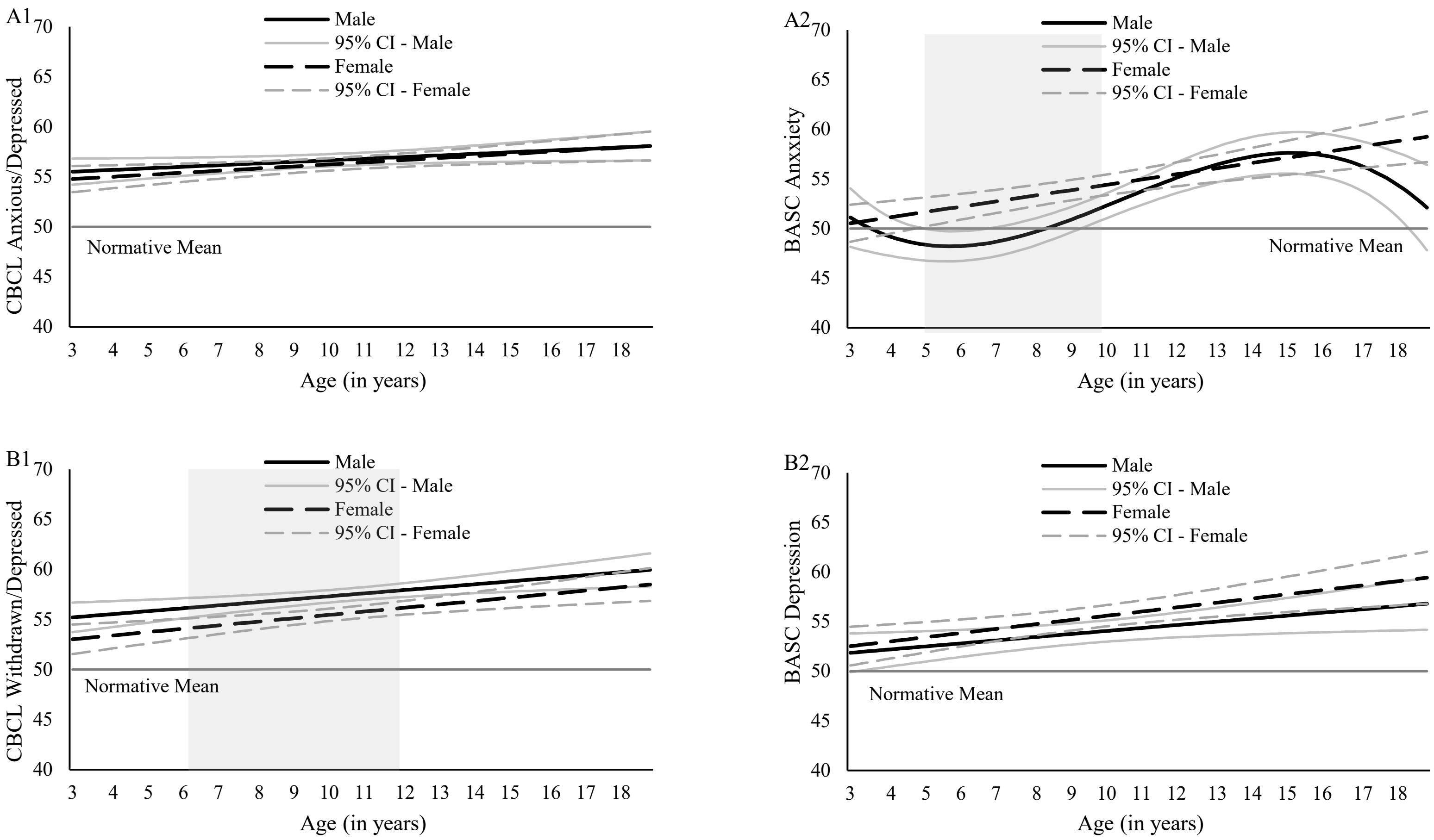

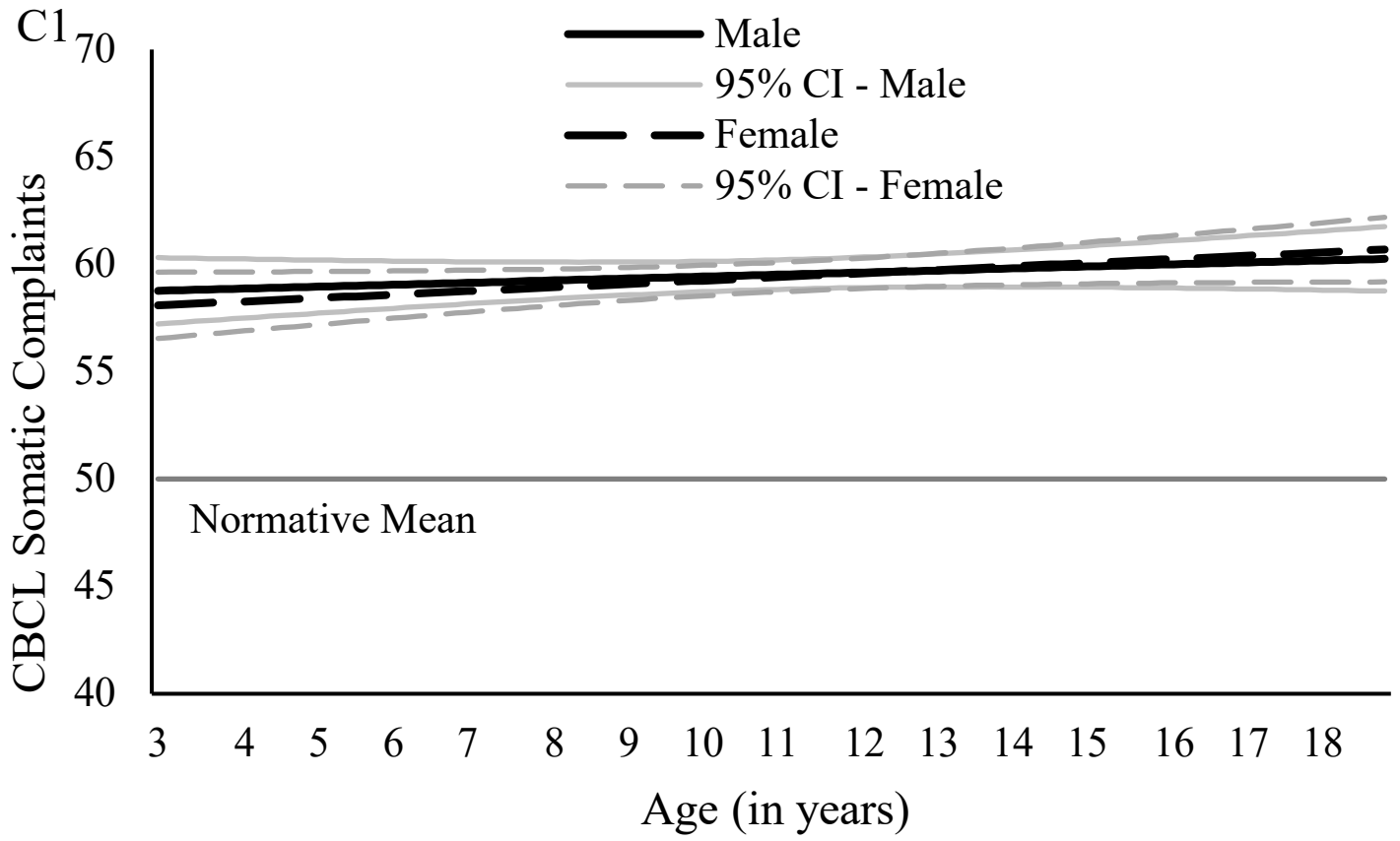

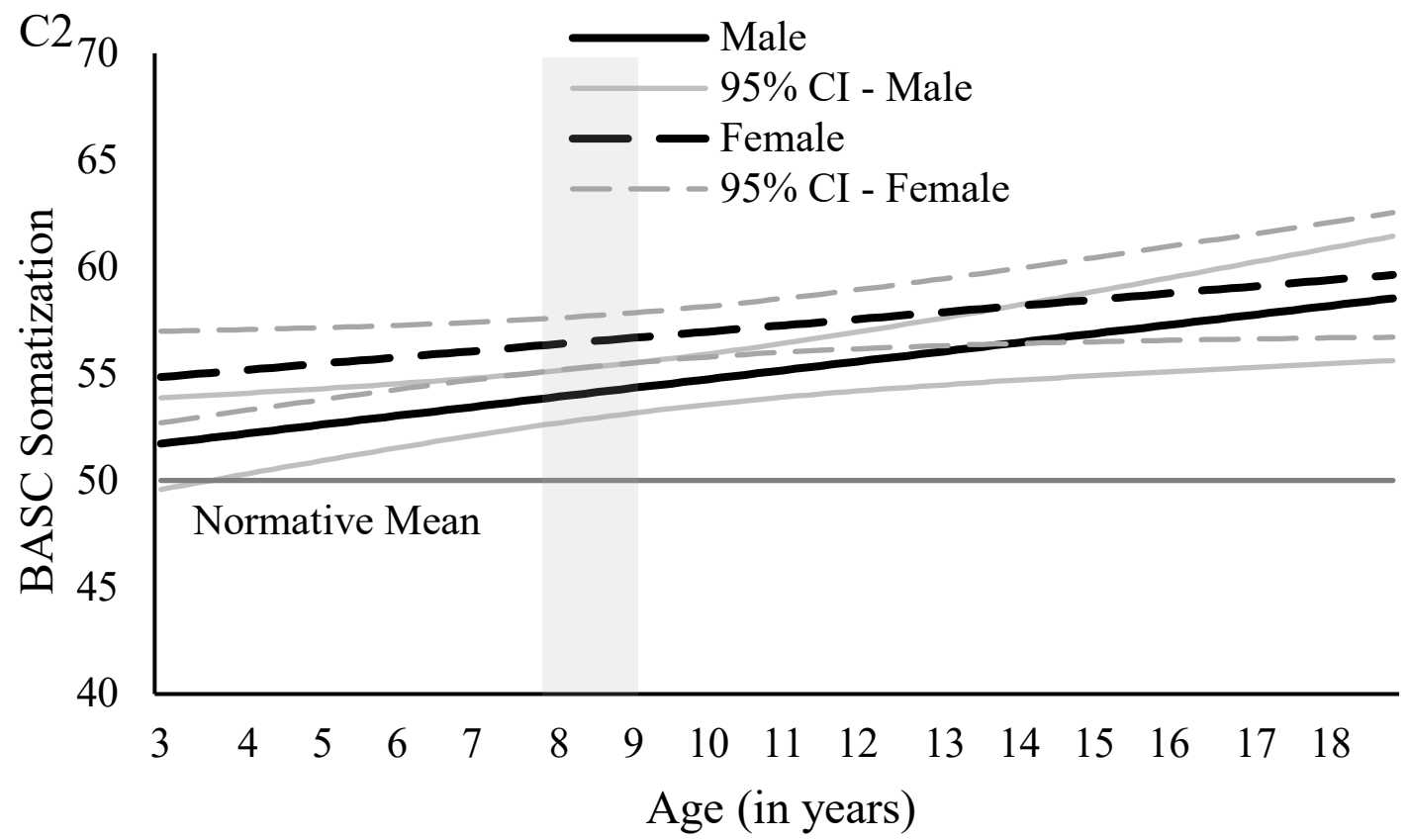

Note. Outcomes include Child Behavior Checklist (CBCL) anxious/depressed (A1), CBCL withdrawn/depressed (B1), CBCL somatic complaints (C1), Behavior Assessment System for Children (BASC) anxiety (A2), BASC depression (B2), and BASC somatization (C2). Internalizing subdomains are presented using standardized T scores (M_{norm} = 50, SD_{norm} = 10). Significant deviations from the normative sample are indicated by 95% confidence intervals (CIs) that do not include the normative mean of 50. Significant group differences are indicated by non-overlapping 95% CIs between specific age points (the shaded grey areas).

eFigure 4. Age Trends of Subdomains of Externalizing Problems across Sex Groups

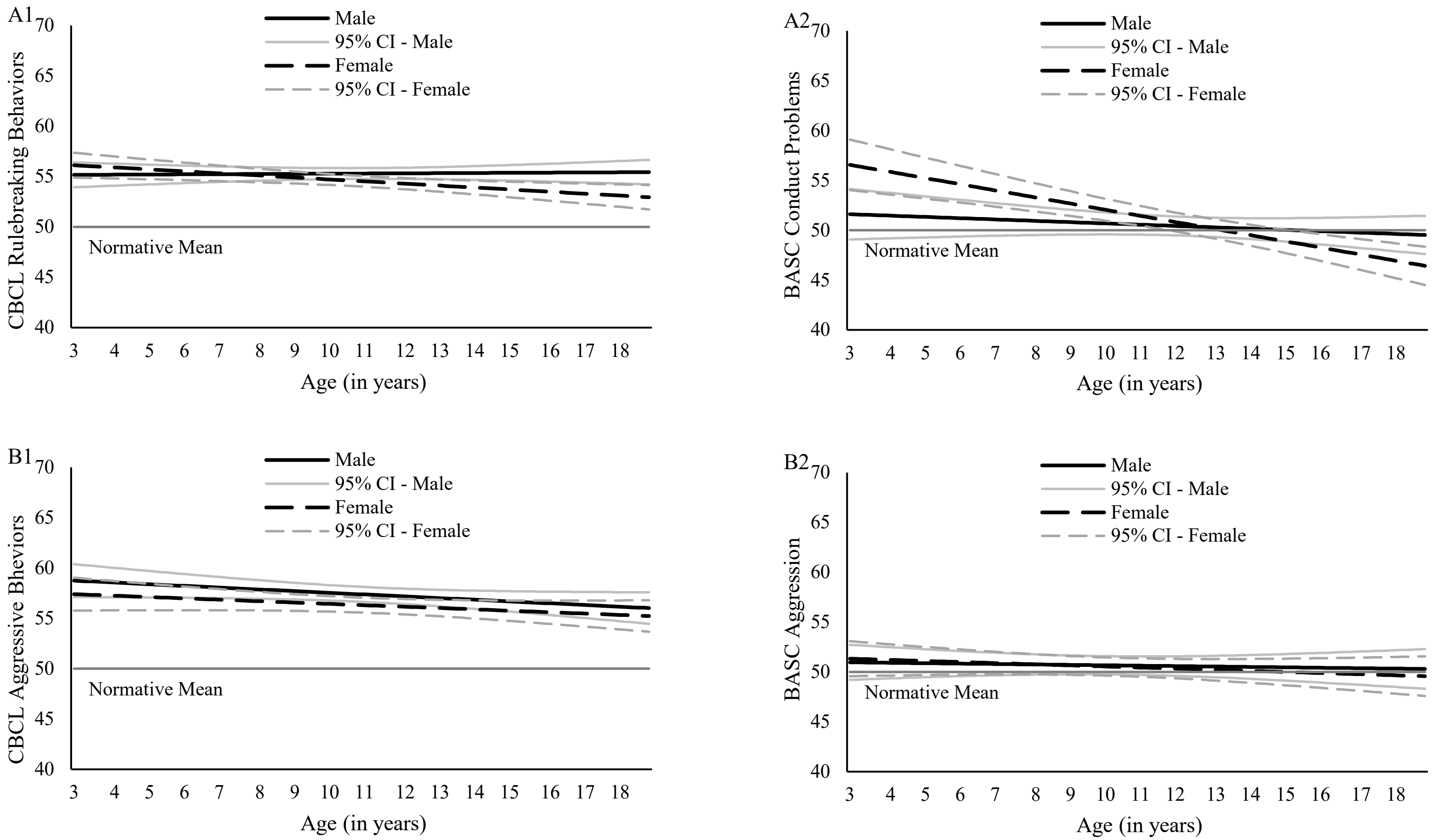

Note. Outcomes include Child Behavior Checklist (CBCL) rulebreaking behaviors (A1), CBCL aggressive behaviors (B1), Behavior Assessment System for Children (BASC) conduct problems (A2) and BASC aggression (B2). Externalizing subdomains are presented using standardized T scores ($M_{\text{norm}} = 50$, $SD_{\text{norm}} = 10$). Significant deviations from the normative sample are indicated by 95% confidence intervals (CIs) that do not include the normative mean of 50. Significant group differences are indicated by non-overlapping 95% CIs between specific age points.

eFigure 5. Age Trends of Subdomains of Internalizing Problems across NF1 Inheritance Mode Groups

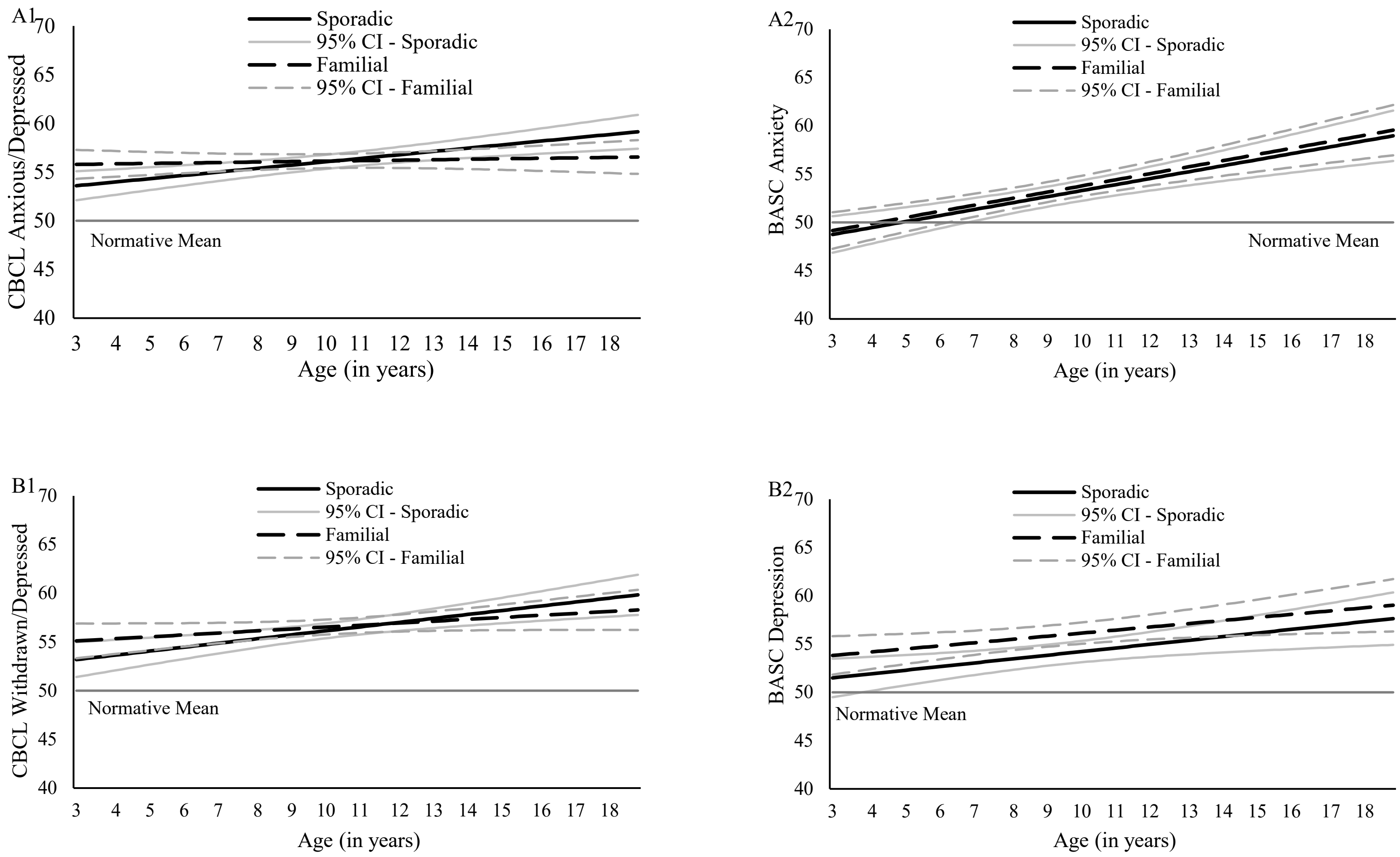

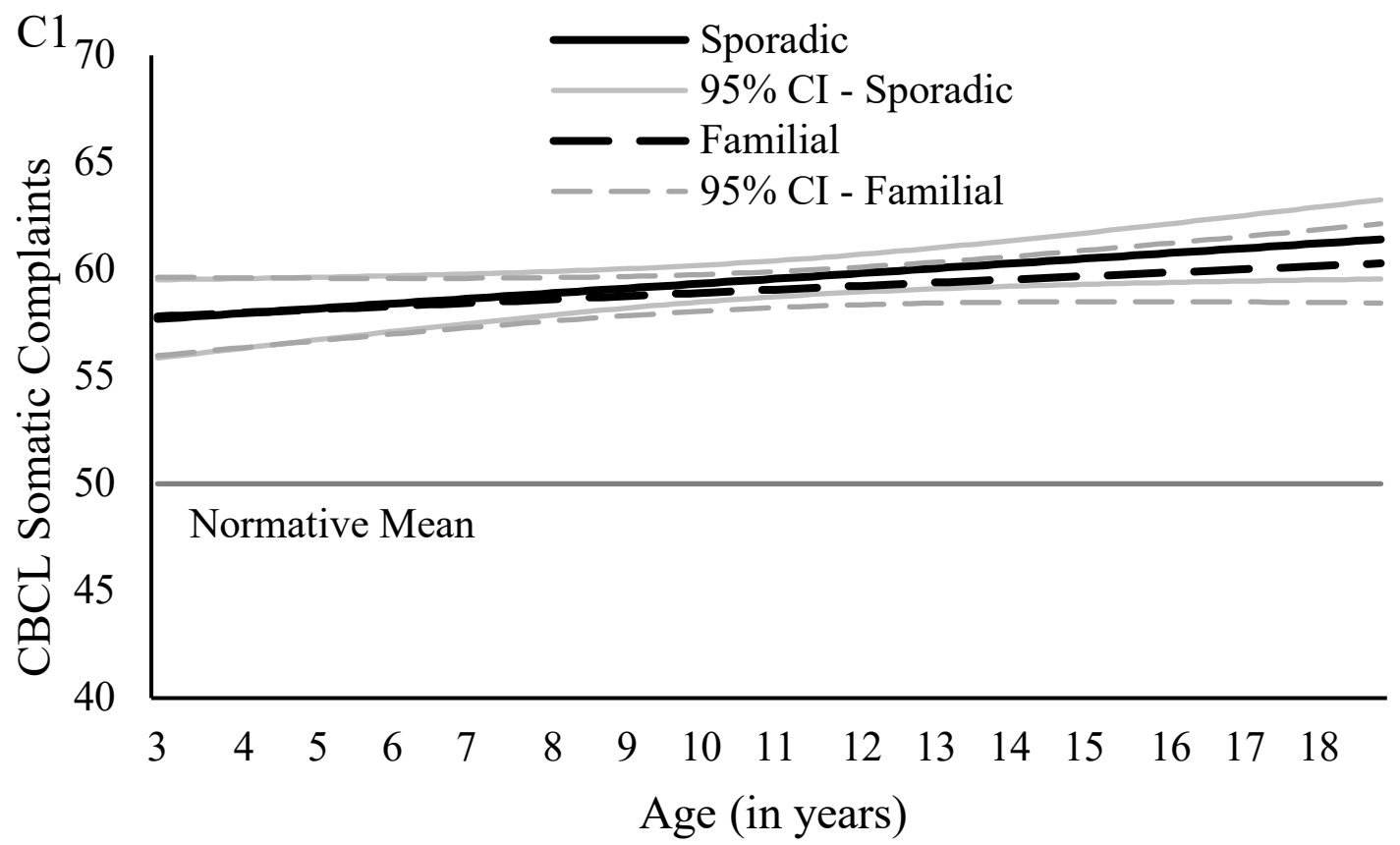

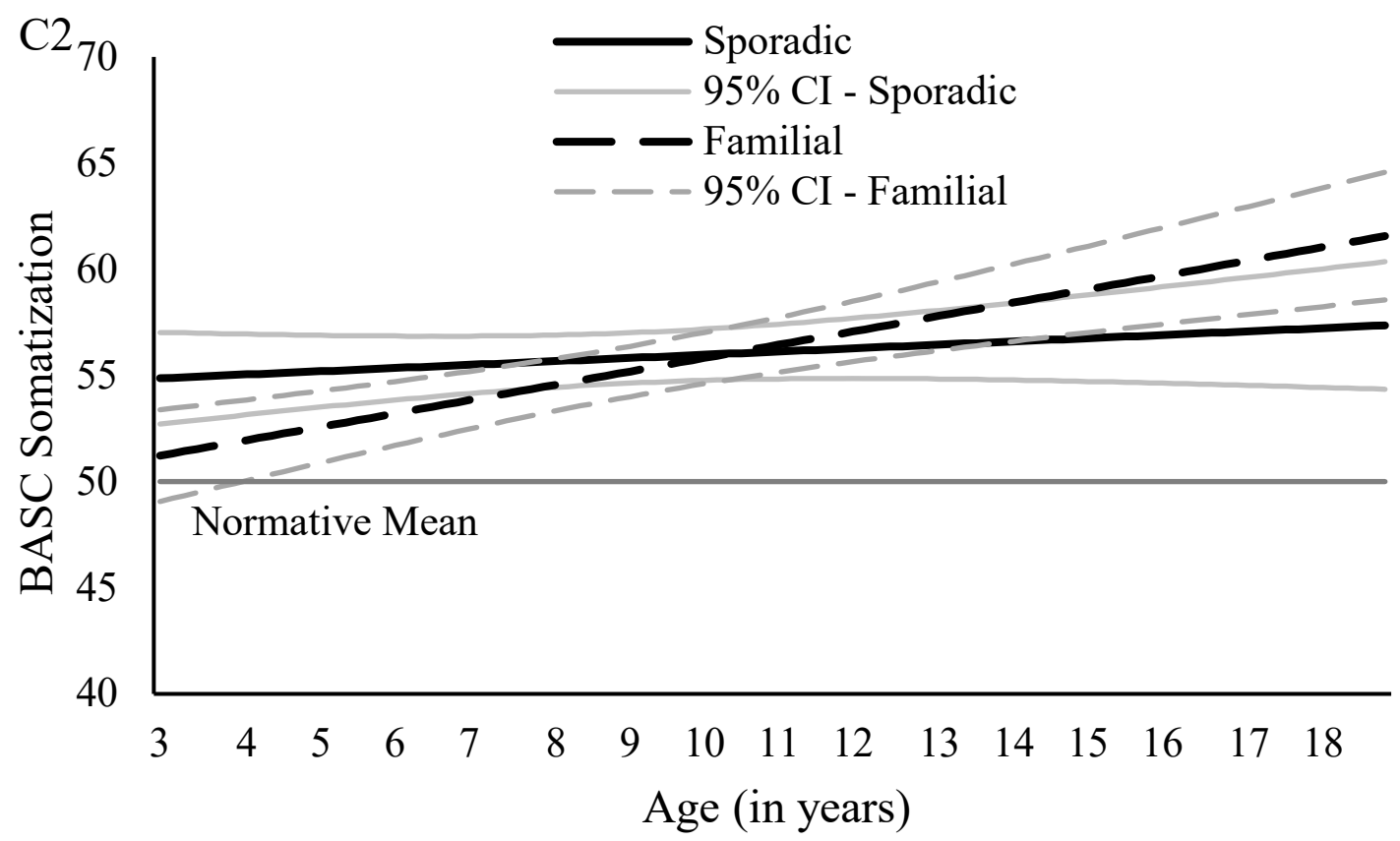

Note. Outcomes include Child Behavior Checklist (CBCL) anxious/depressed (A1), CBCL withdrawn/depressed (B1), CBCL somatic complaints (C1), Behavior Assessment System for Children (BASC) anxiety (A2), BASC depression (B2), and BASC somatization (C2). Internalizing subdomains are presented using standardized T scores (M_{norm} = 50, SD_{norm} = 10). Significant deviations from the normative sample are indicated by 95% confidence intervals (CIs) that do not include the normative mean of 50. Significant group differences are indicated by non-overlapping 95% CIs between specific age points.

eFigure 6. Age Trends of Subdomains of Externalizing Problems across NF1 Inheritance Mode Groups

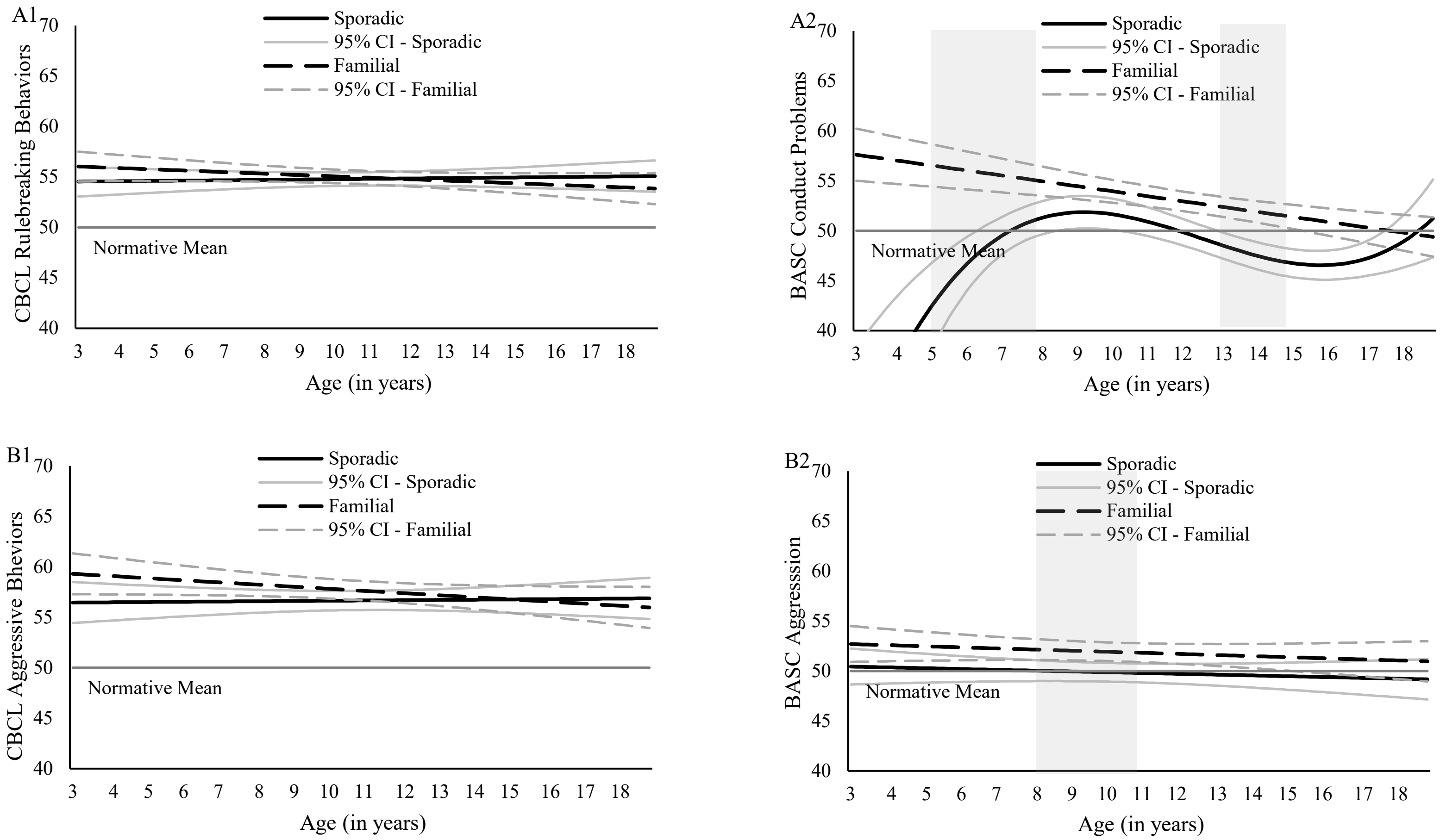

7

Note. Outcomes include Child Behavior Checklist (CBCL) rulebreaking behaviors (A1), CBCL aggressive behaviors (B1), Behavior Assessment System for Children (BASC) conduct problems (A2), and BASC aggression (B2). Externalizing subdomains are presented using standardized T scores (M_{norm} = 50, SD_{norm} = 10). Significant deviations from the normative sample are indicated by 95% confidence intervals (CIs) that do not include the normative mean of 50. Significant group differences are indicated by non-overlapping 95% CIs between specific age points (the shaded grey areas).

eFigure 7. Age Trends of Subdomains of Internalizing Problems across Plexiform Neurofibromas Groups

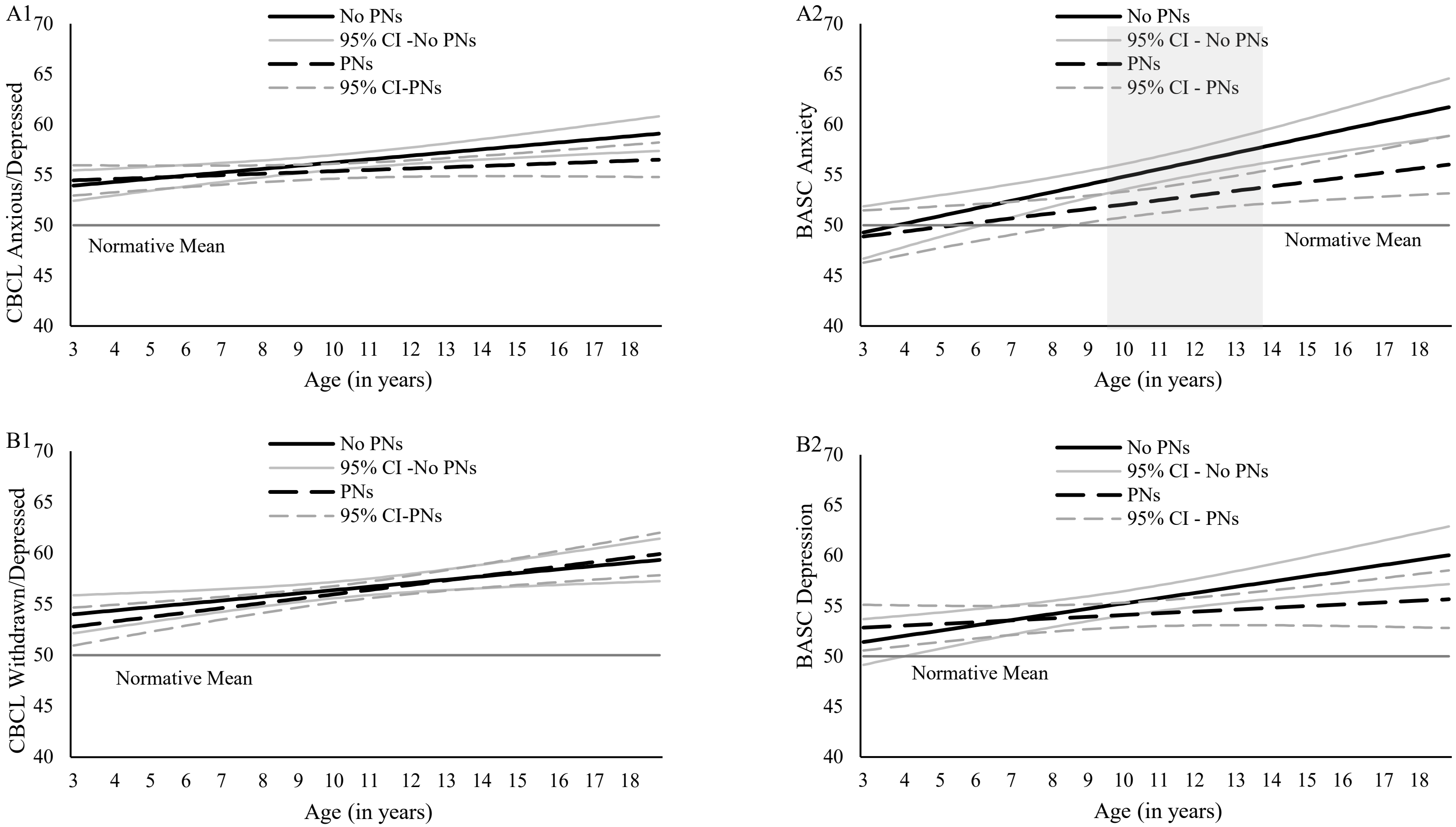

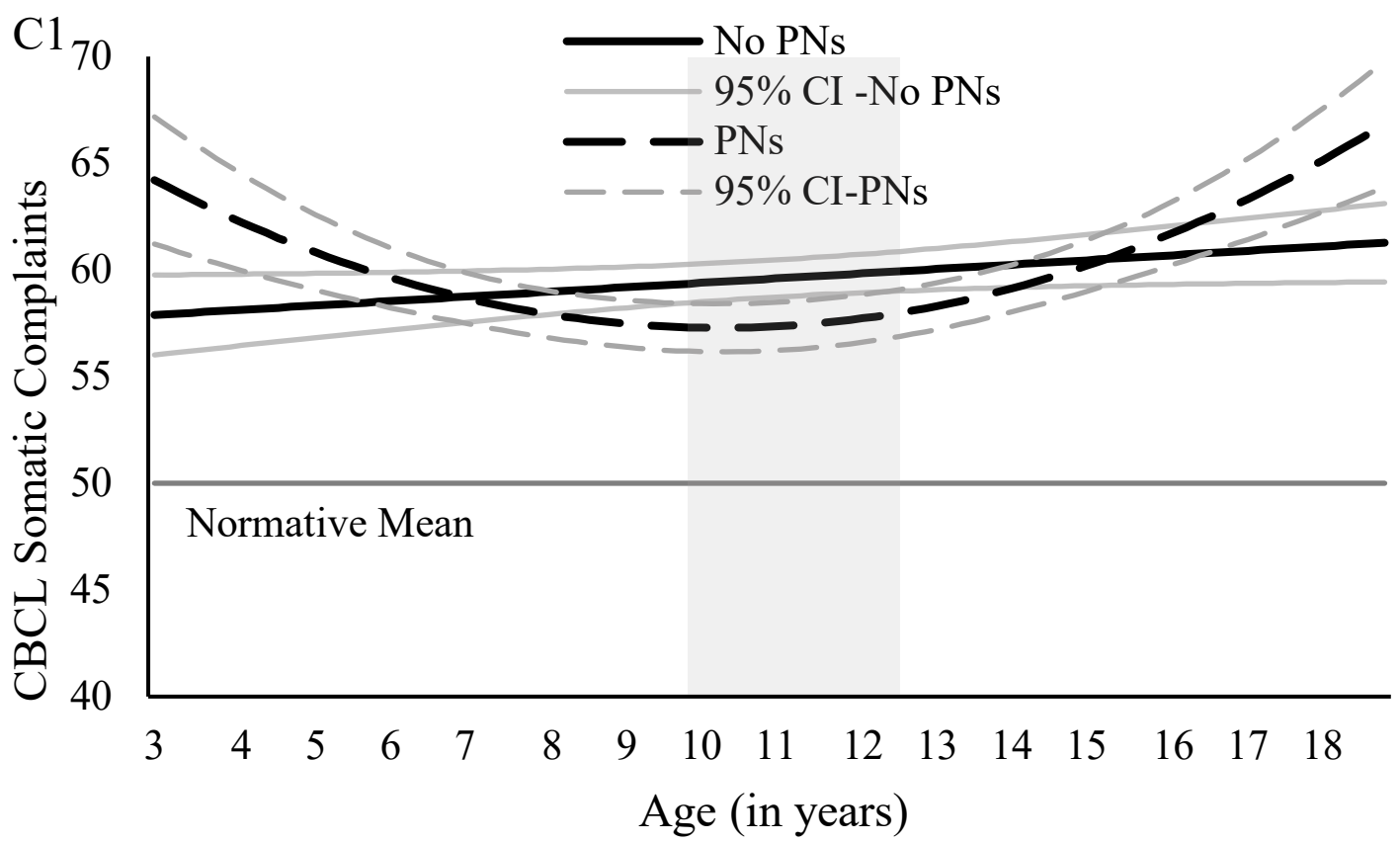

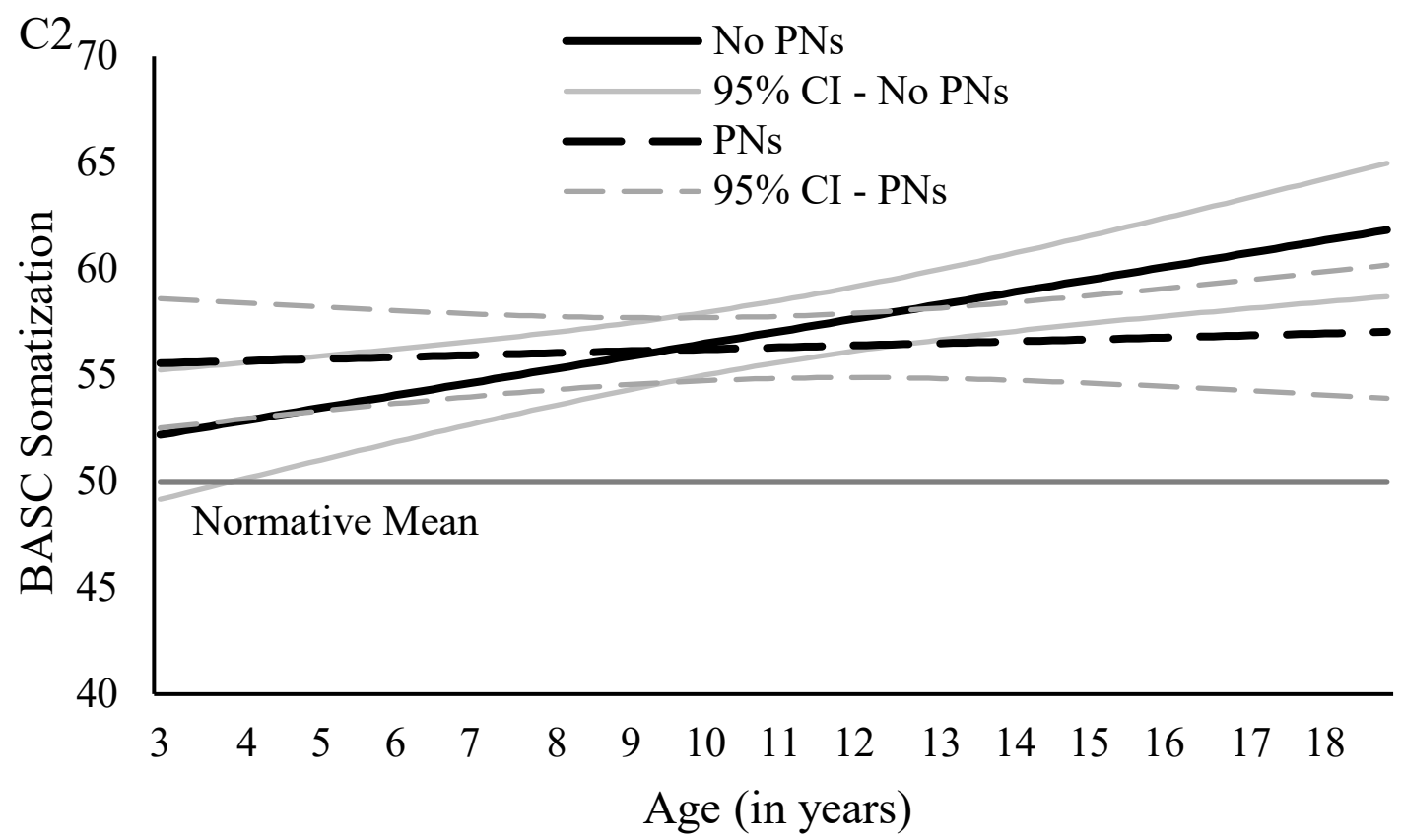

Note. Outcomes include Child Behavior Checklist (CBCL) anxious/depressed (A1), CBCL withdrawn/depressed (B1), CBCL somatic complaints (C1), Behavior Assessment System for Children (BASC) anxiety (A2), BASC depression (B2), and BASC somatization (C2). Internalizing subdomains are presented using standardized T scores (M_{norm} = 50, SD_{norm} = 10). Significant deviations from the normative sample are indicated by 95% confidence intervals (CIs) that do not include the normative mean of 50. Significant group differences are indicated by non-overlapping 95% CIs between specific age points (the shaded grey areas).

eFigure 8. Age Trends of Subdomains of Externalizing Problems across Plexiform Neurofibromas Groups

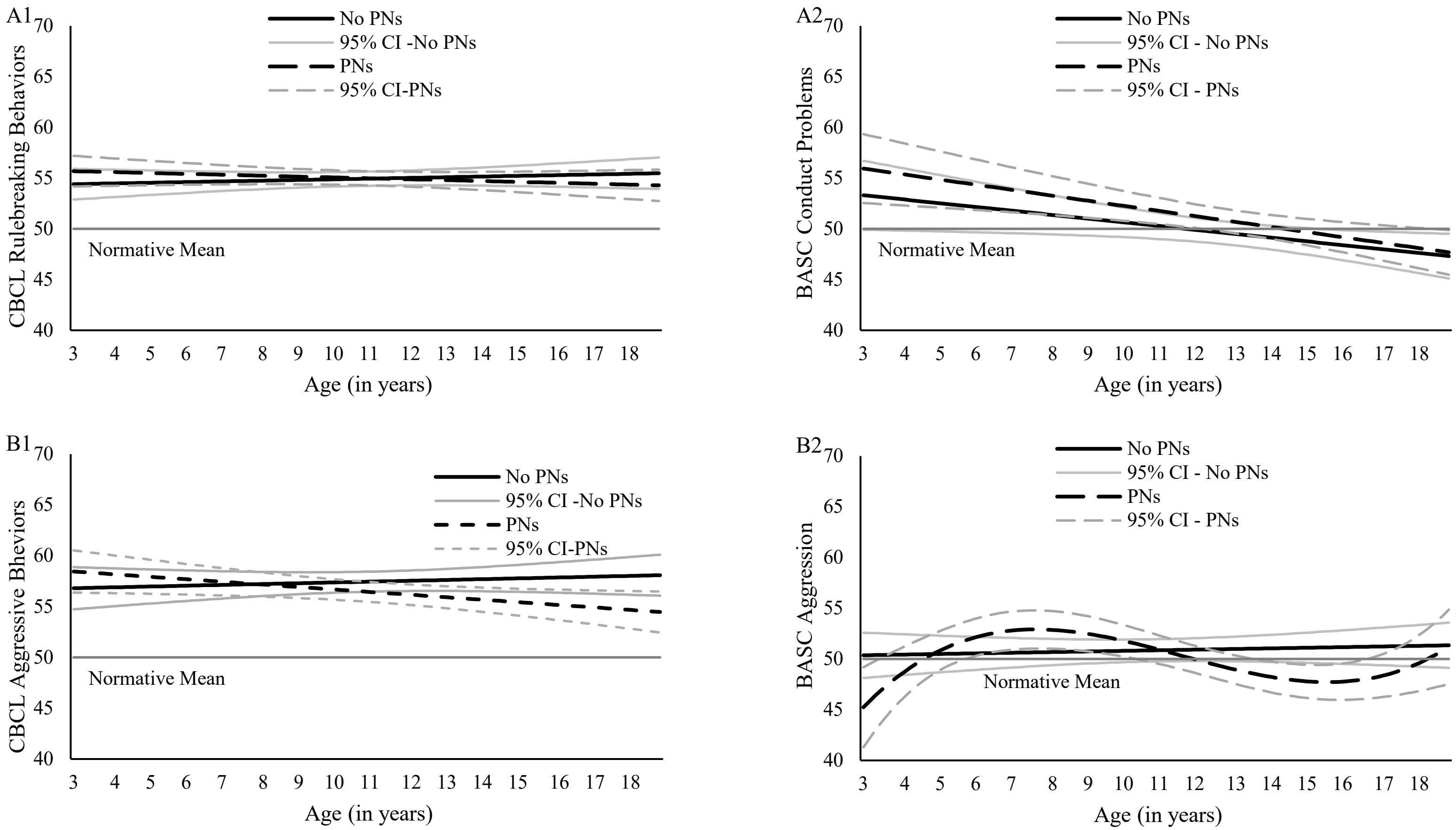

Note. Outcomes include Child Behavior Checklist (CBCL) rulebreaking behaviors (A1), CBCL aggressive behaviors (B1), Behavior Assessment System for Children (BASC) conduct problems (A2), and BASC aggression (B2). Externalizing subdomains are presented using standardized T scores (M_{norm} = 50, SD_{norm} = 10). Significant deviations from the normative sample are indicated by 95% confidence intervals (CIs) that do not include the normative mean of 50. Significant group differences are indicated by non-overlapping 95% CIs between specific age points (the shaded grey areas).

eFigure 9. Age Trends of Subdomains of Internalizing Problems across ADHD Diagnosis Groups

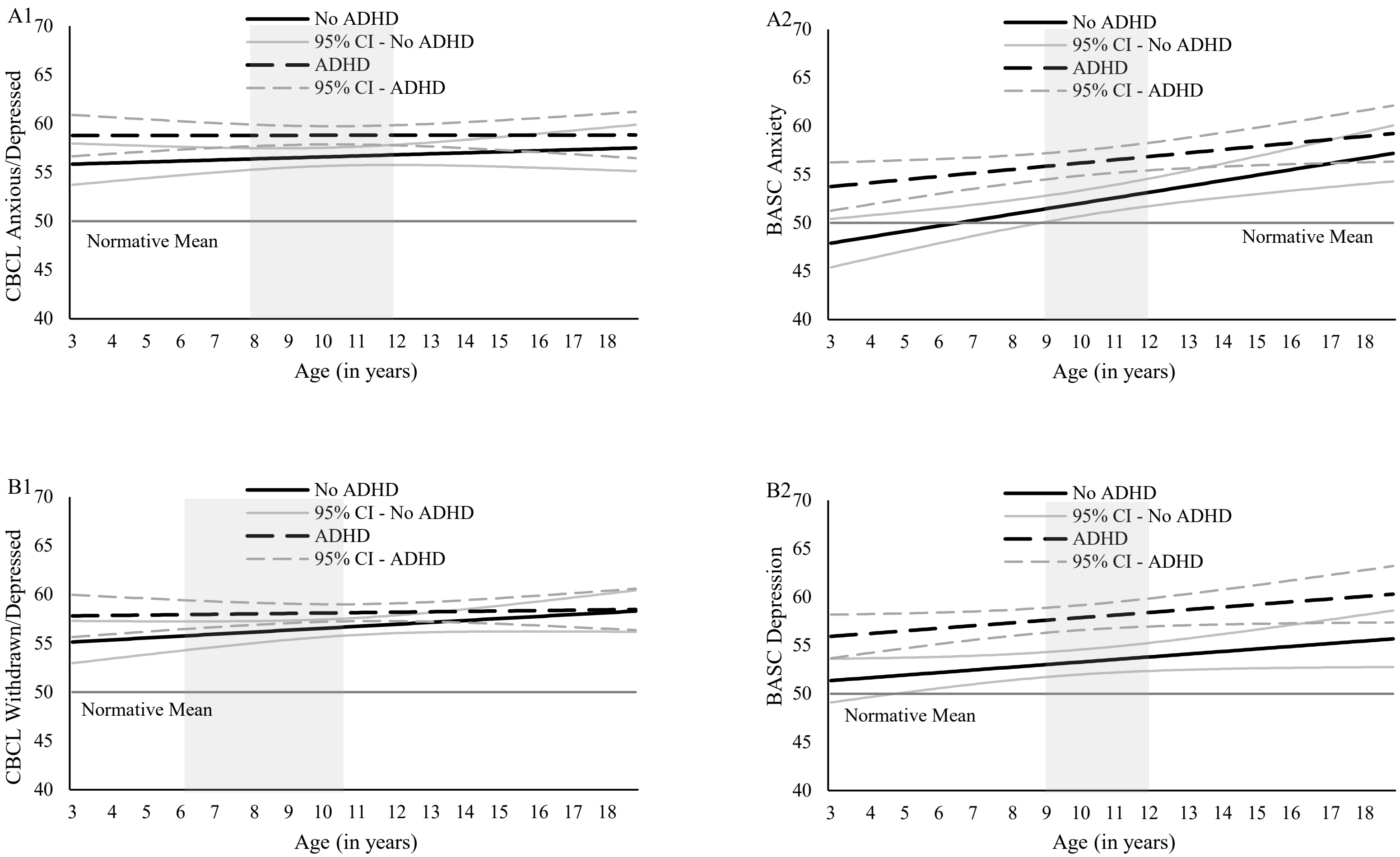

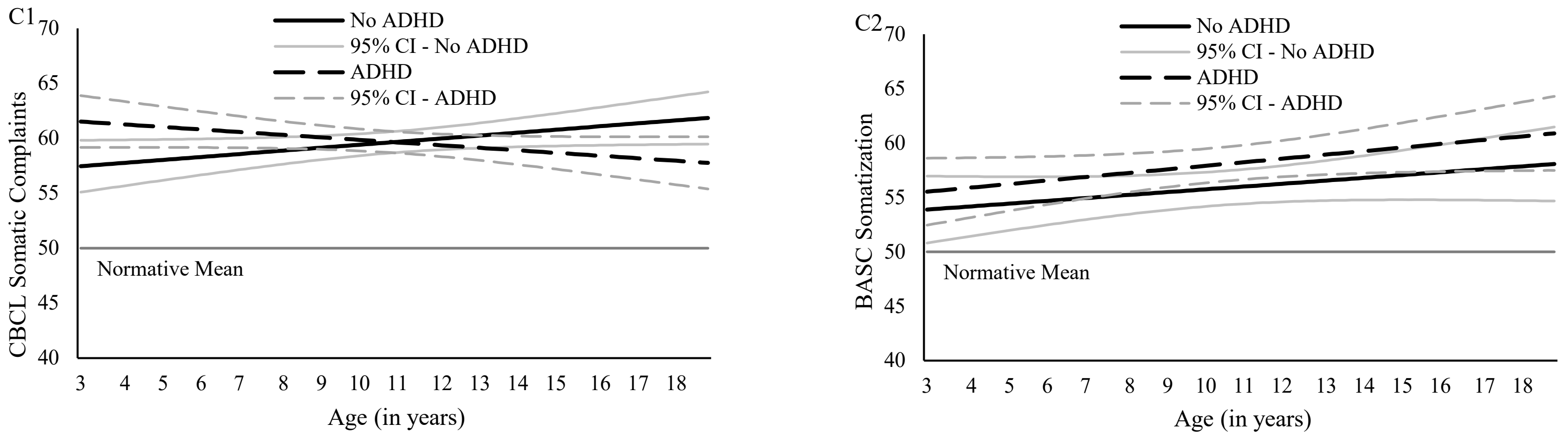

Note. Outcomes include Child Behavior Checklist (CBCL) anxious/depressed (A1), CBCL withdrawn/depressed (B1), CBCL somatic complaints (C1), Behavior Assessment System for Children (BASC) anxiety (A2), BASC depression (B2), and BASC somatization (C2). Internalizing subdomains are presented using standardized T scores ($M_{\text{norm}} = 50$, $SD_{\text{norm}} = 10$). Significant deviations from the normative sample are indicated by 95% confidence intervals (CIs) that do not include the normative mean of 50. Significant group differences are indicated by non-overlapping 95% CIs between specific age points (the shaded grey areas).

eFigure 10. Age Trends of Subdomains of Externalizing Problems across ADHD Diagnosis Groups

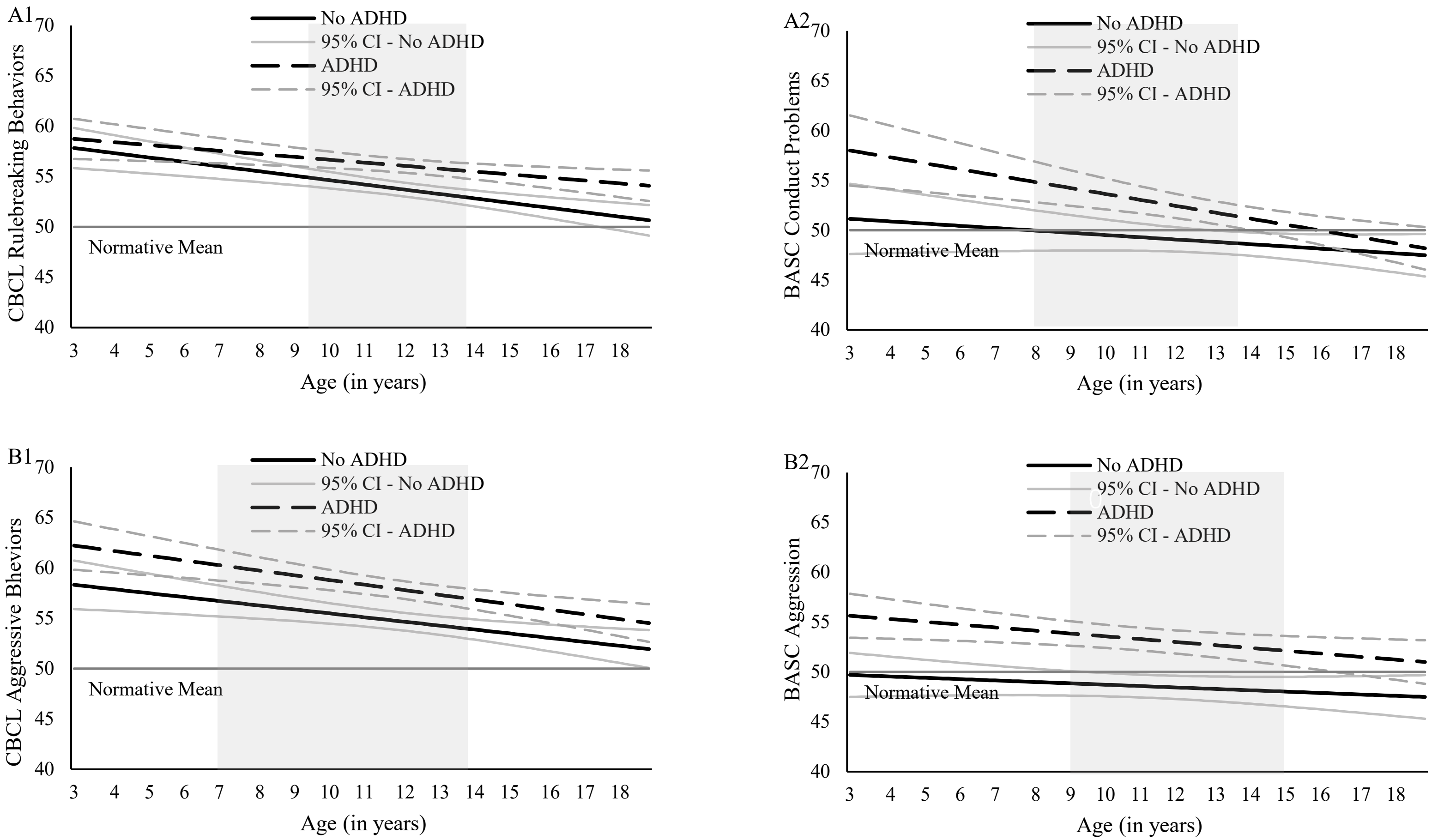

Note. Outcomes include Child Behavior Checklist (CBCL) rulebreaking behaviors (A1), CBCL aggressive behaviors (B1), Behavior Assessment System for Children (BASC) conduct problems (A2), and BASC aggression (B2). Externalizing subdomains are presented using standardized T scores ($M_{\text{norm}} = 50$, $SD_{\text{norm}} = 10$). Significant deviations from the normative sample are indicated by 95% confidence intervals (CIs) that do not include the normative mean of 50. Significant group differences are indicated by non-overlapping 95% CIs between specific age points (the shaded grey areas).
